# Subjectivity and complexity of facial attractiveness

**DOI:** 10.1038/s41598-019-44655-9

**Published:** 2019-06-10

**Authors:** Miguel Ibáñez-Berganza, Ambra Amico, Vittorio Loreto

**Affiliations:** 1 Sapienza University of Rome, Physics Department, Piazzale Aldo Moro 2, 00185 Rome, Italy; 2 Sony Computer Science Laboratories, Paris, 6, rue Amyot, 75005 Paris, France; 3 Complexity Science Hub, Josefstädter Strasse 39, A 1080 Vienna, Austria

**Keywords:** Applied mathematics, Human behaviour

## Abstract

The origin and meaning of facial beauty represent a longstanding puzzle. Despite the profuse literature devoted to facial attractiveness, its very nature, its determinants and the nature of inter-person differences remain controversial issues. Here we tackle such questions proposing a novel experimental approach in which human subjects, instead of *rating* natural faces, are allowed to efficiently explore the face-space and “sculpt” their favorite variation of a reference facial image. The results reveal that different subjects prefer distinguishable regions of the face-space, highlighting the essential subjectivity of the phenomenon. The different sculpted facial vectors exhibit strong correlations among pairs of facial distances, characterising the underlying universality and complexity of the cognitive processes, and the relative relevance and robustness of the different facial distances.

## Introduction

The notions of body beauty and harmony of proportions have fascinated scholars for centuries. From the ancient Greek canons, a countless number of studies have focused on unfolding what is behind the beauty of the face and the body. Nowadays the notion of facial beauty is a fast expanding field in many different disciplines including developmental psychology, evolutionary biology, sociology, cognitive science and neuroscience^1–5^. Still, despite a profuse and multi-disciplinary literature, questions like the very nature of facial attractiveness, its determinants, and the origin of inter-subject variability of aesthetic criteria, elude a satisfactory understanding. Here, we revisit the question drawing conclusions based on an empirical approach through which we allow human subjects to “sculpt” their favorite facial variations by navigating the so called face-space and converging on specific *attractors*, or preferred regions in the face-space.

The face is the part of the human body from which we infer the most information about others, such as: gender, identity, intentions, emotions, attractiveness, age, or ethnicity^6–8^. In particular, looking at a face, we are able to immediately acquire a consistent impression of its attractiveness. Still, we could have a hard time explaining what makes a face attractive to us. As a matter of fact, which variables determine attractiveness and their interactions are still poorly understood issues^3^.

Many works have been devoted to assessing the validity of the *natural selection hypothesis*, or beauty as a “certificate” of good phenotypic condition^7^. According to this hypothesis, a face is judged on average as attractive according to a set of innate rules typical of the human species, which stand out with respect to other social or individual factors. Some degree of consensus has, indeed, been reported^9–13^. Most of these experiments are based on the measurement of correlations among *numerical ratings* assigned to a set of natural (or synthetic^14,15^) facial images by raters belonging to different cultural groups. Much work in this field has also been devoted to assessing the covariation of the perceived beauty of a face with facial *traits* that are believed to signal good phenotypic condition, mainly: facial symmetry, *averageness* and secondary sexual traits. After decades of intense research, the role played by these traits is known to be limited: facial beauty seems to be more complex than symmetry^5^, averageness^14,16^ and secondary sexual traits^7,17^.

Indeed, it has been documented that cultural, between-person and intra-person differences influence attractiveness perception in various ways^4^. As a representative example, the link between masculinity and attractiveness in male faces is subject to significant inter- and intra-subject differences^4,5,7,18^. An evolutionary explanation is that exaggerated masculinity could be perceived as denoting a lack of some personality facets such as honesty or expressiveness^15^. In this context, the so called *multiple fitness* or *multiple motive model*^4,11,19^ proposes that attractiveness varies according to a variety of *motives*, each one evoking a different abstract attribute of the person whose face is evaluated.

On the other hand, an impressive amount of work is committed to the automatic facial beauty rating. This is tackled as a supervised inference problem whose training database is composed of natural facial images codified by *vectors of facial coordinates* in *face-space*^3,20,21^, along with (inter-subject averaged) numerical ratings assigned to them by human subjects, to be inferred. Works differ mainly on the codification of faces in the face-space: from a *geometric* face description (2D or 3D spatial coordinates of the *facial landmarks*), to a detailed description of the *texture* or luminosity degrees of freedom that provide a cue to the facial shape in depth (there also exist *holistic* representations, extracting lower-dimensional, non-local information from the facial image according to some criterion (Principal Component *eigenfaces* or Gabor filters); or using richer techniques which integrate geometric from skin textural and reflectivity characteristics). With the advent of deep hierarchical neural networks, the raw facial image is given as an input to the algorithm, which automatically extracts the putative relevant features in the inference process, although in a hardly accessible way (the *black box problem*).

The supervised inference of ratings may help to address, albeit indirectly, the impact of various facial features on attractiveness. Although the relative relevance of different features has been discussed in various articles, robust conclusions are lacking^3,22–28^. The results about the relative relevance of the *kind* (geometric, textural and holistic) of facial attributes to attractiveness are controversial as well^3,29–33^. In any case, the integration of different kinds of variables seems to improve the inference results^29,34^, suggesting that these are complementarily taken into account in the cognitive process of attractiveness assessment.

Facial beauty is, hence, probably not a universal function of a set of few facial properties, as implicitly assumed in many references, but the result of a complex process in which multiple semantic concepts, providing cues to personality facets, are inferred. The literature concerning inference of personality traits indicates that such semantic concepts may be encoded in global combinations of facial features, in a complex way^35^. This motivates a study of facial beauty beyond the subject-averaged rating, focusing on the inter-subject heterogeneity and on the global combinations of various facial features generating such a diversity.

In summary, the complexity of facial attractiveness perception so far prevented a satisfactory understanding of how attractiveness relates to various facial elements^3^, and of the nature of inter-personal differences. In order to make progress, from a methodological point of view it is important to highlight three key factors. (A) The possible mutual influence among geometric, texture and detailed features^36^. Even considering the problem in terms of geometric variables only, the possible existence of *interactions* or mutual dependencies between different facial components may induce a variety of possible pleasant faces, even for the single subject. (B) The undersampling of the relevant face-space, due to the many different prototypes of facial beauty^14,29^. (C) The subjectivity of the phenomenon, probably hindered by the use of the average numerical beauty ratings. The complexity and richness of the perceptual process, suggested by the multiple-motive hypothesis and by previous work about perception of personality dimensions^6,37–39^, eludes a description in terms of average ratings, a quantity that has already been observed to be inadequate^3^.

In light of these considerations, we here address the phenomenon of facial preference through an empirical approach that aims at removing the biases of ratings, focusing instead on the possibility given to human subjects to freely explore a suitably defined face-space. By means of a dedicated software, based on image deformation and genetic algorithms, we focus on inter-subject differences in aesthetic criterion and let several subjects sculpt their favorite variation of a *reference portrait*, parametrized by a vector of geometric facial coordinates. We observe how different subjects tend to systematically sculpt facial vectors in different regions of the face-space, which we call *attractors*, pointing towards a strong subjectivity in the perception of facial beauty. In addition, the facial vectors sculpted by different subjects exhibit strong correlations for pairs of facial distances, which is a manifestation of the underlying universality and complexity of the cognitive process of facial image discrimination. The correlations contain information regarding the different sources of variability in the dataset of selected vectors. For instance, though a difference between male-female subjects is clearly observed, the largest differences among facial variations, elicited by a principal component analysis, result from criteria that are transversal with respect to the gender only. A third important result concerns the assessment of the robustness of the results with respect to the degrees of freedom not described in the face-space. Crucially, in our approach, the luminance, texture and detailed degrees of freedom are decoupled from the geometric features defining the face-space, and deliberately kept *fixed, and common for all the subjects*. Finally, we observe that the overall experimental results are, interestingly, partially robust and independent of the detailed degrees of freedom (the reference portrait).

The current experimental scheme bypasses the three confounding factors (A–C) mentioned in the precedent paragraph. (A) Uncontrolled sources of biases are absent in our study, since all possible facial variations (given the reference portrait) are described by points in the face-space. (B) In our face-space of reduced dimensionality and unchanged texture degrees of freedom the undersampling is mitigated, making possible an efficient exploration of the face space and allowing for an accurate characterisation of the single-subject attractor. (C) This allow us to fully account for subjectivity: we are able to analyse the differences among different subject’s preferred facial modifications.

## Results

### Preferred facial images as extrema in face-space

**Figure 1:**
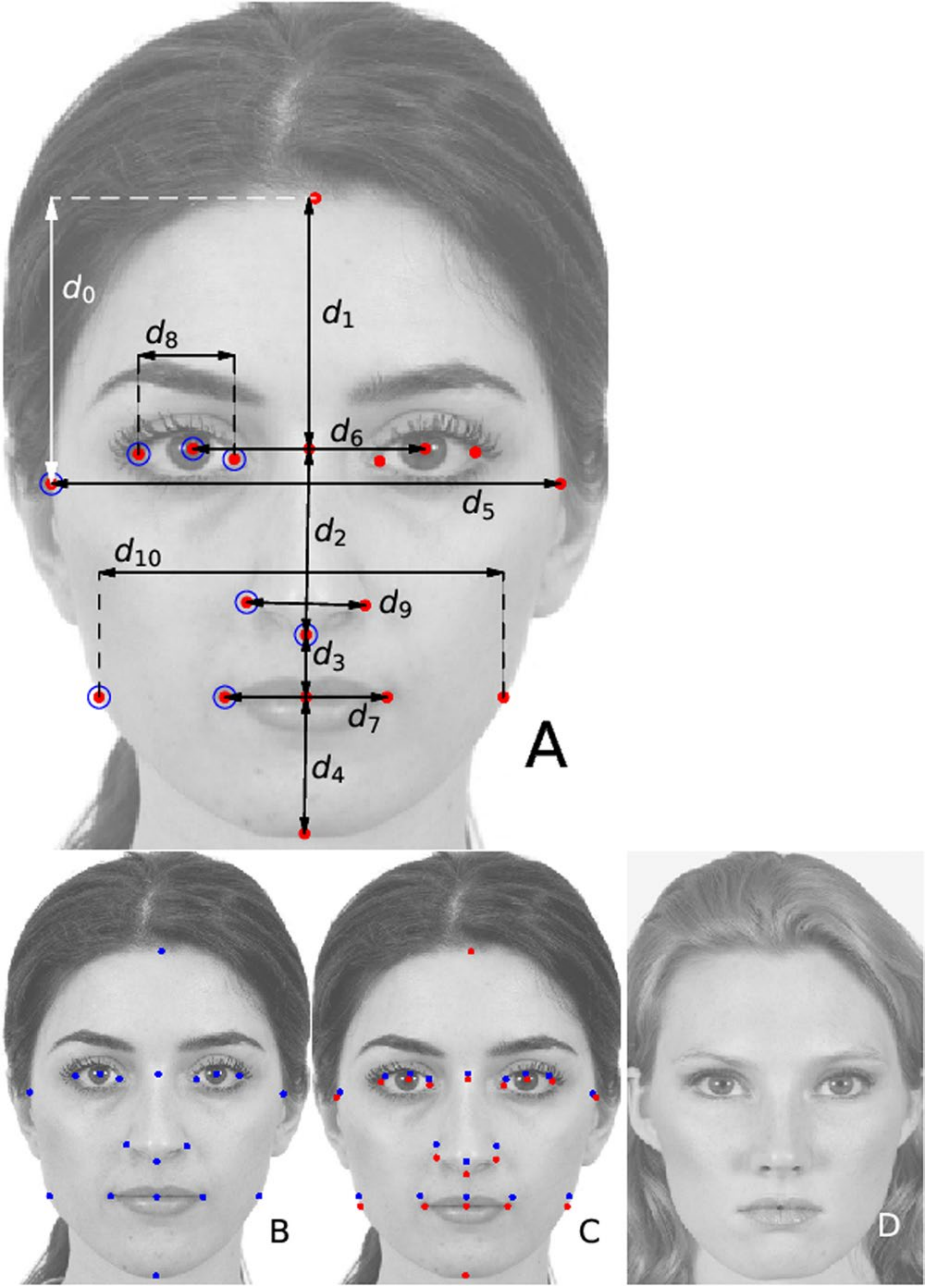
**(A)** The parameters defining the face space. The red points indicate the *landmarks*, *α* = 1, …, 18, whose 2D varying Cartesian coordinates generate the continuum of face space. The face space points are parametrised in terms of vectors **f** whose components are the Cartesian coordinates of a set of non-redundant landmarks ${\overrightarrow{\ell }}_{\alpha }$ (signaled with an empty circle), or in terms of (vertical or horizontal) distances *d*_*i*_ (*i* = 0, …, 10) among some pairs of landmarks ${d}_{i}=|{x}_{\alpha (i)}-{x}_{\beta (i)}|$ or ${d}_{i}=|{y}_{\alpha (i)}-{y}_{\beta (i)}|$ (arrows). **(B)** Reference portrait RP1 used in experiment E1 along with its corresponding landmarks (in blue). **(C)** Image deformation of RP1 according to a given vector of inter-landmark distances **d**: the blue reference portrait landmarks are shifted (leading to the red points) so that their inter-landmark distances are **d**, and the reference image (**B**) is consequently deformed. **(D)** Image deformation of the reference portrait RP2 according to the same vector of distances **d** as in (**C**).

We consider a face-space defined by a set of geometric coordinates illustrated in Fig. 1A. A face is parametrized in terms of a set of 10 non-redundant Cartesian coordinates of 7 single landmarks ${\overrightarrow{\ell }}_{\alpha }=$ (x_*α*_,y_*α*_) or, alternatively, in terms of a vector of *D* = 11 inter-landmark distances ${\bf{d}}={({d}_{i})}_{i=1}^{D}$. The face-space vector components *f*_*i*_ are, in this way, either landmark Cartesian coordinates or inter-landmark distances. From a vector of facial coordinates **f** and a *reference facial portrait* corresponding to a real person, we then construct a facial image by a continuous deformation of the reference portrait such that its landmark geometric coordinates acquire the desired value, **f** (Fig. 1B,C). Within a single experiment, the reference portrait (the image texture) is unchanged and only the geometric position of the landmarks can change (for an in-depth explanation see Sec. Methods and the Supplementary Information).

The aim of the experimental method is to provide a *population* of *N* facial vectors, {**f** ^(*s*,*n*)^}_*n*_, with *n* = 1, …, *N* and ${{\bf{f}}}^{(s,n)}\in {{\mathbb{R}}}^{D}$, for each experimental subject, *s*. Such a population is considered as an empirical sample of the subject’s attractor, or the face-space region of his/her preferred modifications of the reference portrait. This means that the subject would probabilistically prefer facial images associated with vectors that are close to the attractor, rather than local fluctuations away from it (for a precise definition see the Supplementary Section S2). In our experimental scheme, the subject does not sculpt the population by successive discrimination among faces differing by a single coordinate, which turns out to be an inefficient strategy of face-space exploration, but rather through the interaction with a genetic algorithm (see sections Methods, Supplementary Section S3).

**Figure 2:**
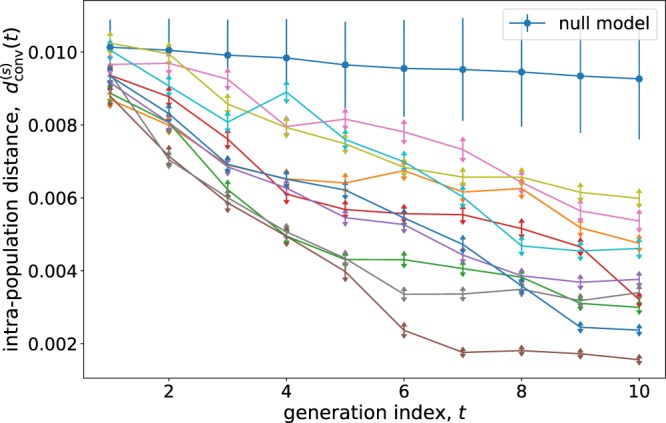
Intra-population distance of the populations sculpted by different subjects (*s*) as a function of the generation (*t*). The Euclidean metrics in face space has been used (see Supplementary Sec. S4), although the results are qualitatively equal for other relevant metrics. Each curve corresponds to a different subject (for 10 randomly chosen subjects). The upper curve of joined circles corresponds to the null model genetic experiment, in which the left/right choices are random.

In a first experiment (E1), we have let *S*_1_ = 95 subjects sculpt their facial variations of reference portrait RP1 (see 1-A). This results in a final population, ${{\mathscr{S}}}_{1}={\{{{\bf{f}}}^{(s,n)}\}}_{s=1,n=1}^{{S}_{1},N}$ of *N* = 28 facial vectors for each subject. Starting from *N* initial random facial vectors, the FACEXPLORE software generates pairs of facial images that are presented to the subject, who selects the one that he/she prefers. Based on *N* left/right choices, a genetic algorithm produces a successive generation of *N* vectors, in a constant feedback loop of offspring generation and *selection* operated by the subject. The iteration of this process leads to a sequence of *T* generations of facial vectors, each one more adapted than the last to the subject’s selection criteria, eventually converging to a pseudo-stationary regime in which the populations are similar to themselves and among consecutive generations. Figure 2 reports the evolution (versus the generation index, *t* = 1, …, *T* = 10) of the *intra-population distance*, the distance among faces within the single populations sculpted by 10 different, randomly chosen, subjects in E1 (see Supplementary Section S4 for details). In the next subsection, we discuss the degree of reproducibility of our results as a function of *N*, *T* and *S*_1_.

The intra-population distance decreases with the generation index, indicating that the populations sculpted by single subjects tend to clusterize in a region of the face-space. This clustering is not observed in a null experiment in which the left-right decisions are taken randomly. Remarkably, a diversity of behaviors towards the pseudo-stationary regime is observed, already signaling differences in the way the face-space is explored.

From now on, we will consider the final population sculpted by the *s*–th subject, ${\{{{\bf{f}}}^{(s,n)}\}}_{n=1}^{N}$, as the final, *T* = 10-th generation of the sequence of populations sculpted by this subject in E1. In the next subsection we show that the face-space attractors of different subjects are actually significantly and consistently different. This experimental scheme is, therefore, able to *resolve* the subjective character of attractiveness, as the single subject tends to sculpt populations of vectors clustered in a narrow region in the face-space in successive realisations of the experiment. All these facts imply that the single subject attractor can be operationally characterised as an *extremum of a subject-dependent, probabilistic function in face-space*, which may be inferred from the populations sculpted by the subject in several instances of the experiment (see Supplementary Section S2 for a complete definition). The attractors are extrema of such a function in the sense that a significant fluctuation of a vector coordinate away from its value in the attractor will tend to lower its probability of being selected by the subject, given the reference portrait.

### Assessment of subjectivity: distinguishable aesthetic ideals

In order to assess the subjectivity of the sculpting process, we need to measure to what extent the same subject, by repeating the same experiment, would sculpt populations of facial vectors closer to each other than to populations sculpted by distinct subjects. To this end we performed a second experiment (E2), in which a subset of *S*_sc_ = 6 subjects were asked to perform *m* = 6 instances of an experiment E1, with the common reference portrait RP1, different (random) initial conditions and sequence of random numbers in the genetic algorithm. The subjectivity is assessed through the comparison of two sets of distances: (i) the (*S*_sc_*m*(*m* − 1)/2) *self-consistency distances* among facial populations sculpted by the same subject in different instances of the experiment E2; (ii) the (*S*_1_(*S*_1_ − 1)/2) *inter-subject distances* between couples of populations sculpted by different subjects in experiment E1 (see Supplementary Section S4 for details). If subjectivity was at play in the sculpting process, and not hindered by the stochasticity of the algorithm, the self-consistency distances would be lower than inter-subject distances.

**Figure 3:**
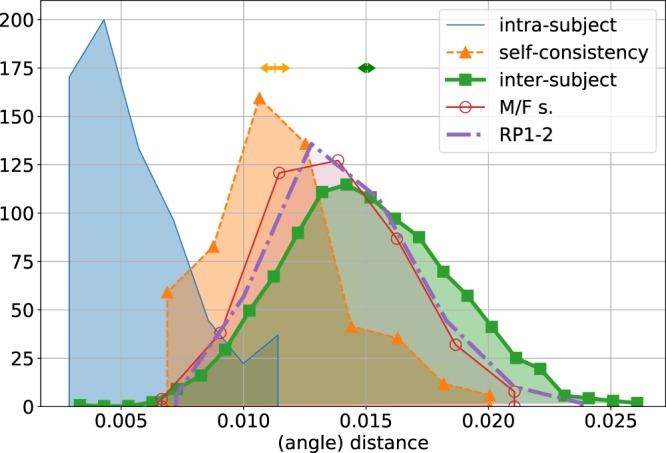
Main panel: Normalised histograms of pseudo-distances. **Blue**: *subject intra-population distances*, or self-distances of all the populations sculpted in E1. **Orange**: *self-consistency distances*, or distances among couples of populations sculpted by the same subject in E2. **Green**: *inter-subject distances*, or distances among couples of populations sculpted by different subjects in E1. **Purple**: distances among couples of populations sculpted by different subjects in different experiments, E1 and E3 (differing in the reference portrait). **Red**: distances among couples of populations sculpted by subjects of different gender in E1. The orange and green arrowed segments over the self-consistency and inter-subject histograms indicate the confidence intervals of the histogram averages, ${\mu }_{{\rm{sc}}}\pm {\sigma }_{{\rm{sc}}}/{n}_{{\rm{sc}}}^{\mathrm{1/2}}$ and ${\mu }_{{\rm{i}}}\pm {\sigma }_{{\rm{i}}}/{n}_{{\rm{i}}}^{\mathrm{1/2}}$ respectively, with *n*_sc_ = *S*_sc_*m*(*m* − 1)/2 and *n*_i_ = *S*_1_(*S*_1_ − 1)/2.

This is clearly the case, see Fig. 3: self-consistency distances are lower than inter-subject distances (Student’s *p* < 10^−30^). In Fig. 3 we also report the histogram of *intra-population distances*, i.e., the average distance among the vectors belonging to a population, for different populations scuplted by different subjects in E1 (blue curve). The intra-population distances are not suitable for an assessment of the subject self-consistency, since they strongly depend on the number of generations performed by the genetic algorithm (c.f. Fig. 2). The emerging scenario is that of single subjects who, in a single realization of the sculpting experiment, end up in a very clustered population (blue curve in Fig. 3). Performing several realizations of the same experiment leads the subject to a slightly different population in face-space (orange curve in Fig. 3, labelled “self-consistency”). These self-consistent populations are anyway closer to each other than to populations sculpted by different subjects, as witnessed by the larger *inter-subject distances*, whose histogram is presented in the green curve in Fig. 3. A crucial point is that the distance between the inter-subject (green curve, i) and self-consistency (orange curve, sc) histograms in Fig. 3, $t=({\mu }_{{\rm{i}}}-{\mu }_{{\rm{sc}}})/({\sigma }_{{\rm{i}}}^{2}+{\sigma }_{{\rm{sc}}}^{2}{)}^{\mathrm{1/2}}=\mathrm{0.82(1)}$ (see Supplementary Fig. S3) would be *even larger* in an experiment with a higher number of generations *T*. Using larger values of the genetic algorithm parameters *T* and *N* would result in a lower value of *μ*_sc_, at the cost of a larger experimental time, since *NT* binary choices are required from the subject (see Sec. Methods and Supplementary Sec. S3). Furthermore, larger values of *S*_1_, *S*_sc_, *m* would give rise to a lower statistical error of the considered observables (see Supplementary Sec. S4), proportional to $1/\sqrt{{S}_{\mathrm{1,}{\rm{sc}}}}$ and, in particular, to an even more significant difference among both histograms, since the uncertainty of their average is proportional to *σ*_i_/*S*_1_ and ${\sigma }_{{\rm{sc}}}/\sqrt{{S}_{{\rm{sc}}}m}$, respectively. In any case, the values used in experiments E1-2 are large enough to assess the differences among different subjects’ attractors in a significant way.

The set of populations ${{\mathscr{S}}}_{1}={\{{{\bf{f}}}^{(s,n)}\}}_{s,n}$ sculpted in E1 exhibits facial coordinates which vary in a wide range: roughly 0.018(10) per coordinate of the total face length, corresponding to ~3.2 mm in the average female face^40^ (see the average 〈**f**〉 and standard deviation **σ** of the single coordinates in Supplementary Fig. S5). The self-consistency distance *μ*_sc_ ± *σ*_sc_, with which the experiment allows to resolve the single-individual attractor is, remarkably, much lower, equal to 0.0067(18) per coordinate (using the simple Euclidean-metrics in face-space, see Supplementary Section S8), barely twice the pixel image resolution, ~400^−1^ (Figure Supplementary Section S4). This quantity corresponds to 1.18(30) mm in the female average facial length.

Several metrics among facial vectors have been used to compute the inter-subject and self-consistency distances: *Euclidean, Mahalanobis, angle-* and *Byatt-Rhodes metrics* (see Supplementary Section S4 and^20,21^). The angle-metrics (the angle subtended among standardised Principal Components (PC’s) in face-space) turns out to be the one with which the statistical distinction is more significant (see Supplementary Fig. S3, and subsection “Differences induced by the subject gender” for the definition of PC’s). This result is compatible with previous work proposing that such face-space metrics is the one that best captures differences in facial identity^21,41^. Further results regarding the *t*-value difference among both histograms as a function of the face-space metrics can be found in the Supplementary Section S4. Using the simple Euclidean metrics (the Euclidean distance per coordinate in physical coordinates), the inter-subject and self-consistency distances result slightly more overlapping, although still clearly distinct. For the sake of the statistical discernibility among the inter-subject and self-consistency distances, it is observed that the 10 dimensions involved in the definition of the face space are redundant in the sense that defining the face-space metrics in terms of the 7 most varying PC’s, the two sets of distances result more significantly different (see Supplementary Fig. S3). For completeness, in Fig. 3 we also report two further sets of distances. The red line histogram corresponds to pseudo-distances among pairs of populations sculpted by subjects of different gender in E1, while the purple line histogram corresponds to the pseudo-distances among pairs of populations sculpted by different subjects with different reference portraits (E3, see “Relevance of facial features”, before).

These findings highlight the intrinsic subjectivity of facial attractiveness. Despite the limited freedom of choice, the reduced dimension of the face-space, and the common reference portrait, single subjects tend to sculpt a region of face-space that is systematically closer to their previous selections than to other subjects’ sculptures. Indeed, the probability of two facial vectors sculpted by the same subject to be closer than two facial vectors sculpted by different subjects in E1 is *p*_12_ = 0.79(1) (see Supplementary Section S8).

A further interesting observation about Fig. 3 concerns the overlap between the histograms of *self-consistency* and *inter-subject distances*. Its existence allows us to reconcile the strong subjectivity unveiled by experiments E1-2, and the universality reported in the literature. The couples of facial vectors which are involved in distances for which there is a high overlap correspond to commonly preferred faces, around the most probable vector in the dataset, 〈**f**〉. Within a low experimental precision, or an accuracy larger than the standard deviation per coordinate *a* > |***σ***|/*D*, all the subjects appear to agree in their choices. Under this perspective, the reported universality of beauty could be the side-effect of an experimental procedure where subjects express their preferences among a limited set of predefined options, the real facial images, in a high-dimensional face-space (indeed, the effective number of relevant facial dimensions may be of the order of hundreds^42^). In such an undersampling situation, different natural faces exhibit very different number of facial coordinates *g*_*i*_ (or, more precisely, of PC’s, see before), close to the most probable value 〈*g*_*i*_〉, with respect to their standard deviation (say, *σ*(*g*_*i*_)). The faces exhibiting many coordinates in the commonly preferred region are consensually preferred, and most highly rated^20^. By letting the subjects sculpt instead their preferred modification in a lower-dimensional face space, as in experiments E1-2, the subjects exclude extreme values of the coordinates, and manage to fine-tune them according to their personal criterion. In this circumstance, it is possible to resolve the subjects’ preferences with higher accuracy, *μ*_sc_ < |***σ***|/*D*, unveiling a strong subjectiveness. Our data suggest that the higher the accuracy with which the single subject attractor is resolved, the more distinguishable different subjects’ attractors result in the face space. This picture suggests a *complete subjectivity*, or complete distinctiveness of different subjects’ criteria (see also Sec. Methods).

### Correlations among different facial features

In our experimental scheme, only geometric degrees of freedom may change. This allows us to determine the personal attractors efficiently and accurately, in a not too high-dimensional face-space. Moreover, it avoids the uncontrolled influence of features not described in the face-space. However, as anticipated in Sec. Introduction, it is also essential in this framework to account for possible mutual dependencies between different components of the facial vectors.

Besides the average and standard deviation of single coordinates referenced above, a quantity of crucial importance, despite the scarce interest that the literature has dedicated to it, is the correlation among facial coordinates from subject to subject. We denote with **y** the standardised fluctuations of the vector **f** around the experimental average, ${y}_{i}=({f}_{i}-\langle {f}_{i}\rangle )/{\sigma }_{i}$. The sculpted facial vectors presenting a fluctuation of a coordinate *y*_*i*_ (say, a larger mouth width, *y*_7_ > 0 in terms of inter-landmark distances) *tend to consequently present positive and negative fluctuations of other facial coordinates y*_*j*≠*i*_ (e.g., a higher mouth, *y*_4_ > 0). The sign and magnitude of such covariations is given by the *correlation matrix* among fluctuations of facial coordinates. This is the positive definite, symmetric matrix ${C}_{ij}=\langle {y}_{i}{y}_{j}\rangle$, averaged over subjects $\langle \cdot \rangle ={\sum }_{s=1}^{S}\cdot \,/S$. In order to subtract the influence of correlations within the single-subject attractor, only one population vector, of index *n*_*b*_(*s*), uncorrelated and randomly distributed, is considered for each subject *s*; the average and standard deviation of the matrix elements *C*_*ij*_ have been obtained from many bootstrapping realisations, labelled by *b*, of the indices *n*_*b*_(*s*), see Supplementary Section S4. The experimental matrix *C* exhibits a proliferation of non-zero elements (32% of the matrix elements presenting a *p*-value < 5 · 10^−2^, see Supplementary Section S11), unveiling the presence of strong correlations among several couples of facial coordinates.

The most strongly correlated *C* elements are among vertical or horizontal distances (see Supplementary Fig. S9 and Table S4). Such strong correlations are easily interpretable: wider faces in ${{\mathscr{S}}}_{1}$ tend to exhibit larger inter-eye distances and wider mouths and jaws; higher nose endpoints, in their turn, covary with higher mouths and eyes; higher eyes covary with higher mouths, and so on. Perhaps the most remarkable aspect of the matrix *C* is the proliferation of couples of vertical-horizontal coordinates, highlighting the crucial role played by *oblique* correlations. The sign of oblique correlations *C*_*ij*_ (see Supplementary Table S4) is such that fluctuations of a landmark position ${\overrightarrow{\ell }}_{\alpha }$ covary with fluctuations of different landmarks ${\overrightarrow{\ell }}_{\beta }$ in such a way some inter *α*,*β*-landmark segment slopes are restored with respect to their average value. This is so for the most correlated couples of vertical-horizontal coordinates *i*, *j* (*p* < 5 · 10^−2^).

**Figure 4:**
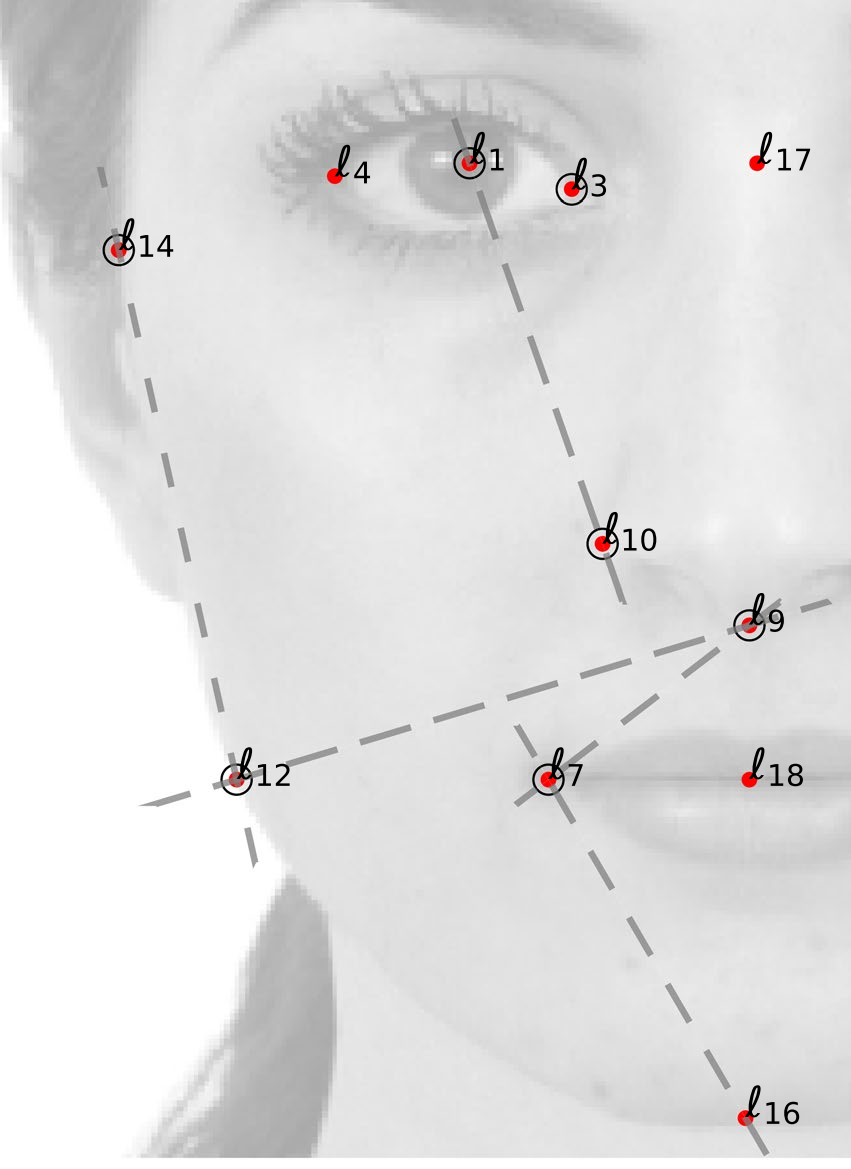
Relevant inter-landmark segments. The correlation matrix elements *C*_*ij*_ involving vertical and horizontal landmark coordinates, $\langle {x}_{\alpha (i)}{y}_{\alpha (j)}\rangle$ can be understood geometrically as a statistical invariance of the value of some inter-landmark segment slopes (dashed lines) with respect to their average value (represented in the figure). The sign of oblique *C*_*ij*_’s coincide with that of the slope of the inter-landmark lines $(\langle {y}_{\alpha (i)}\rangle -\langle {y}_{\alpha (j)}\rangle )/(\langle {x}_{\alpha (i)}\rangle -\langle {x}_{\alpha (j)}\rangle )$. For instance, the most correlated horizontal-vertical landmarks are 〈x_12_y_9_〉, exhibiting a positive sign (c.f. Supplementary Table S4): indeed, for lower nose endpoints (which correspond to a positive fluctuation y_9_ > 〈y_9_〉), the 9–12 angle can be restored only by increasing the x_12_-coordinate, x_12_ > 〈x_12_〉.

The information brought by the correlation matrix helps in this way to construct a remarkably clear picture of the experimental distribution of facial vectors. The inter-subject differences and the experimental stocasticity induce fluctuations around the average facial vector **y** = **0**. The fluctuations are, however, strongly correlated in the facial coordinates, in such a way that vertical and horizontal coordinates covary positively and, *at the same time*, the value of some inter-landmark segment slopes shown in Fig. 4, of prominent relative importance, do not change too much with respect to their average value (see Supplementary Section S13).

These findings indicate that, for a meaningful inference of the perceived attractiveness in face-space, one should consider the impact of at least *linear combinations* of facial coordinates, rather than the impact of single facial coordinates. The intrinsic complexity of attractiveness perception cannot be satisfactorily inferred through a simple regression of facial datasets using a sum of functions of single facial coordinates (see also Supplementary Section S14 and^43^).

### Relevance of facial features: the variable hierarchy

In this section we discuss the robustness of the results presented above. One of the crucial questions in facial attractiveness is what is the relevant set of variables which mainly determine the perceived attractiveness of a face^3,36^. A formulation of the problem in theoretical-information terms is that of finding a hierarchy of relevant facial features. It is such that, when enriching the description with more variables in high levels of the hierarchy, the resulting variables in lower levels result unchanged. In the present study, the geometric quantities can be considered as low-level variables in the extent to which they are not influenced by the reference portrait, or by the luminance and texture facial features that have been disregarded and kept unchanged in the face-space description.

To settle this question, we performed a third experiment, dubbed E3, in which we asked the *S*_1_ participants in E1 to repeat the experiment using a different reference portrait (RP2, see Fig. 1D). Afterwards, we have compared the resulting set of sculpted facial vectors, ${{\mathscr{S}}}_{3}$, with the outcome of experiment E1, ${{\mathscr{S}}}_{1}$. Interestingly, a statistical *t*-test shows that, while some facial coordinates result clearly distinguishable, others result statistically indistinguishable, signifying their robustness with respect to the texture facial features determined by the reference portrait. These are, in terms of inter-landmark distances, *d*_*i*_, the coordinates *d*_2,6,7,10_, indistinguishable with *p* > 0.1 (see Supplementary Fig. S6). If, instead of focusing on the distribution of single quantities *y*_*i*_, one considers instead the correlations, *y*_*i*_*y*_*j*_, the results (see Supplementary Table S4) turn out to be robust within their statistical errors, since only 2% of the matrix elements *C*_*ij*_ result significantly distinguishable (*p* < 0.075, and none of them for *p* < 0.05).

The ensemble of these results implies a strong robustness of the results presented above, namely the subjectivity and the correlations among different facial features, with respect to a change in the reference portrait. It is remarkable that the coordinates *i* = 2, 6, 7, 10 in ${{\mathscr{S}}}_{1}$ are indistinguishable from those in ${{\mathscr{S}}}_{3}$ up to a remarkably small scale. For them, the average difference of couples of coordinates, ${\langle {f}_{i}^{(s)}-{f}_{i}^{(s{\rm{^{\prime} }})}\rangle }_{s,s{\rm{^{\prime} }}}$ (with subjects *s*, *s*′ belonging to E1 and E3, respectively), vanishes up to small fluctuations, lower than the statistical error of such quantity. Such an error, of order (*S*_1_*S*_3_)^−1/2^, see Supplementary Section S10, is: $\sigma ({f}_{i}^{(s)}-{f}_{i}^{(s{\rm{^{\prime} }})})=1.54\cdot {10}^{-2}$ per coordinate, which corresponds to 0.27 mm in the average female face. We consider this result as one of the most remarkable of the present work. It highlights the striking robustness of the inter-landmark distances *d*_2,6,7,10_. Such variables are, therefore, in low levels of the variable hierarchy, suggesting that they have prominent and intrinsic importance in the cognitive mechanism of face perception.

### Differences induced by the subject gender

An extensively debated question in the literature is to what extent the subject gender influences attractiveness, a question that the present experimental scheme is particularity suited to address. Partitioning the dataset accordingly, ${{\mathscr{S}}}_{1}={{\mathscr{S}}}_{{\rm{m}}}\cup {{\mathscr{S}}}_{{\rm{f}}}$, it is obtained that, again, some facial coordinates are barely distinguishable or completely indistinguishable in both sets (*d*_3,4,6,7_, see Supplementary Fig. S7). Conversely, some coordinates are noticeably distinguishable. Compared to female subjects, male subjects tend to prefer thinner faces and jaws (*d*_5,10_), lower eyes (*d*_1_), higher zygomatic bones (*d*_0_), larger eye width (*d*_8_). The difference becomes very distinguishable along *d*_2,9_ (*p* < 3 · 10^−3^, Supplementary Fig. S7): males definitely prefer shorter and thinner noses. These results are partially in agreement with previous findings in the literature, that highlight male subjects’ preference for smaller lower face area and higher cheekbones^14,44^. Furthermore, they also provide accurate relative differences along each coordinate and reveal that, at least for the two reference portraits RP1-2, the facial feature leading to larger differences among men and women attractors *is the nose*.

A deeper insight is obtained by the analysis of PC’s. These are the projections of the physical coordinates on the *C*-matrix eigenvectors, **y**′ = *E***y** (where $EC{E}^{\dagger }={\rm{diag}}({\lambda }_{1},\ldots ,{\lambda }_{D})$). The different principal components ${y^{\prime} }_{i}$ are, in other words, uncorrelated linear combinations of the physical coordinates ($\langle {y^{\prime} }_{i}{y^{\prime} }_{j}\rangle ={\lambda }_{i}{\delta }_{ij}$). Principal components corresponding to large eigenvalues (as ${y^{\prime} }_{10}$) represent the linear combinations of physical coordinates accounting for as much of the database variability, while those corresponding to the lowest eigenvalues represent the most improbable, or “forbidden” linear combinations of fluctuations away from the average **y** = **0** (see the Supplementary Information). Different principal axes (**e**^(*k*)^, the rows of matrix *E*) describe the different, independent sources of variability in the dataset, that could reflect the subjects’ traits most distinguishing their aesthetic criteria (as the gender).

**Figure 5:**
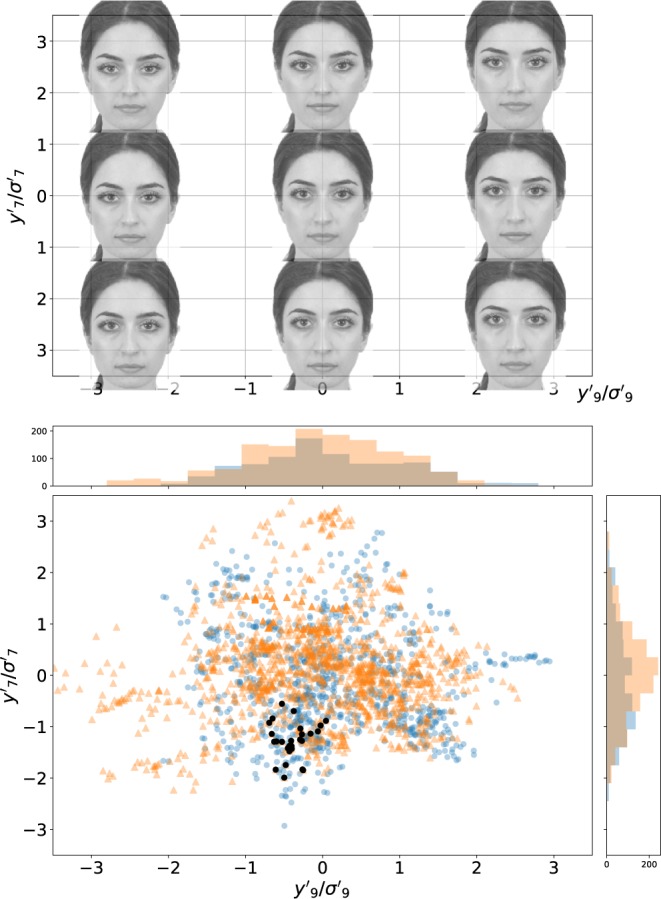
Top figure: facial images corresponding to the deformation of the average facial vector along two different principal axes (the **e**^(7)^, **e**^(9)^ eigenvectors of the correlation matrix *C*, corresponding to the fourth and second larger eigenvalues, *λ*_7_, *λ*_9_). The axes represent the principal components along these axes, (*y*′_9_, *y*′_7_) in units of their standard deviations (${\lambda }_{i}^{\mathrm{1/2}}$). In other words, the image is generated from the facial vector $y={E}^{\dagger }({y^{\prime} }_{7}{{\bf{e}}}^{\mathrm{(7)}}+{y^{\prime} }_{9}{{\bf{e}}}^{\mathrm{(9)}})$. Bottom figure: selected facial vectors. Each point is a projection of a selected facial vector in the principal axes corresponding to the Top figure, i.e., each point has coordinates ${y^{\prime} }_{7}^{(s,n)},{y^{\prime} }_{9}^{(s,n)}$, for all *s*, *n* in the E1 dataset. Blue points correspond to male subjects, and orange triangles to female subjects (male subjects tend to sculpt vectors with ${y^{\prime} }_{9} < 0$, and vice-versa). The black points correspond to a population sculpted by a single, randomly selected, subject.

It turns out that faces corresponding to different subject’s gender are distinguishable on three PC’s (see Supplementary Fig. S8). Quite interestingly, such principal axes are not the ones exhibiting the largest eigenvalue, suggesting that the largest differences among selected faces correspond to inter-subject *criteria* that are transversal with respect to the subject’s gender. Figure 5 shows some image deformations of the average face along two principal axes: **e**^(9)^, **e**^(7)^ (the 2nd and the 3rd most variant eigenvectors of *C*). The PC defined by **e**^(9)^ is male/female distinguishable (males preferring negative values of *y*′_9_). Instead, the *y*′_7_ coordinate is gender-indistinguishable, and it could correspond to a different subject’s quality, as the predilection for assertiveness, neoteny, or a different personality dimension, in the language of the *multiple motive hypothesis*^4,11,19^.

## Discussion

In this article, we have introduced an experimental behavioural method that allows human subjects to efficiently select their preferred modification of a reference portrait in the multi-dimensional face-space (and, in principle, in general spaces of images that can be parametrised with 2D landmark coordinates). The method allows to flexibly and accurately determine the face-space regions which are representative of a given subject’s criterion. It opens the path to a novel, data-driven approach to cognitive research in face perception, allowing scholars to: (1) quantitatively address the inter-subject differences *in the resulting sculpted shapes, beyond the rating*; (2) isolate the influence of a *secondary* set of variables (such as texture features) and *a posteriori* address their influence (something that cannot be directly done with databases of natural facial images); (3) analyse a resulting set of facial vectors without being limited or conditioned by the *a priori* correlations present in natural image databases.

The method (based on our software FACEXPLORE, whose details are explained in the Supplementary Information) permits a highly accurate description of the single subject or subject category preferences in the face-space, thanks to the geometric/texture separation of facial degrees of freedom and to a genetic algorithm for efficient search in the face space. Using this technique, we have performed a set of experiments in which the single subjects preferred region in the face space have been determined with an unprecedented accuracy, below the millimeter per facial coordinate.

Such experiments allow us to draw the following conclusions. First of all, attractiveness turns out to be associated with the existence of subject-dependent specific regions in the face space that we dubbed attractors, highlighting the essential subjectivity of attractiveness. Despite the limited face-space dimension, and the homogeneity of the statistical universe (composed of subjects of the same cultural group), different subjects clearly tend to prefer different facial variations, suggesting that the subjectivity should be taken into account for a complete scientific picture of the phenomenon. Larger databases and more heterogeneous statistical universes would only make the essential subjectivity of attractiveness perception even more evident.

In light of these facts, the validity of the natural selection hypothesis (universality, impact of averageness, symmetry and sexually dimorphic traits) may be arguably a matter of the precision of the length scale and of the facial image resolution of the facial description. Within a sufficiently accurate description of the subjects’ criterion in face-space, the phenomenon emerges in its whole complexity, showing that the preferred faces of different subjects are systematically different among themselves and, consequently, different from the average face. In their turn, these differences reflect personal features and circumstances that condition the subject’s preferences, one of which is the subject’s gender.

The second important conclusion we can draw concerns the patterns associated to different subjects’ attractors. Different sculpted facial vectors exhibit strong correlations among pairs of facial distances, characterising the underlying universality and complexity of the cognitive processes, leading, in its turn, to the observed subjectivity^4^. Our study reveals, in particular, the crucial importance of correlations among vertical and horizontal coordinates, whose existence and relevance have been, to the best of our knowledge, only postulated^22,24,35^. Different facial variations are strongly correlated, a fact that confirms the holistic way in which we perceive faces^36^. Our results suggest to consider attractiveness not as a scalar quantity, rather as the outcome of a complex process in which various semantic motives are evaluated. These are probably encoded in pairwise and higher-order correlations among facial features, more than in the value of single facial coordinates^35^.

A third result concerns the role of the subject’s gender in the assessment of attractiveness. This is, indeed, an important source of diversity in our dataset. Nose length and width, eye height, face and jawbone width, zygomatic bone height, turn out to be the main facial traits distinguishing male and female observers. However, a principal component analysis suggests that the largest differences among selected facial variants correspond to principal axes that are independent of the subject’s gender. Abstract personality dimensions have been observed to be consensually attributed to faces, and the impact of such qualities on various facial elements have been measured through principal component analysis^6,37–39^. Such principal axes could be correlated with those of the present study. This would be a confirmation of the postulated connection between attractiveness and personality judgments^1,6,45^. It would allow to elicit the different traits that are judged by the subjects in a bottom-up, data-driven fashion.

A further noticeable result is the assessment of the influence of the reference portrait in the distribution of sculpted facial vectors. Quite remarkably, the *a priori* dimensionality reduction implicit in our analysis (ignoring texture degrees of freedom), turns out a posteriori to be sufficient and justified (see Sec. Methods).

In summary, the novel experimental approach proposed in this article allowed us to unveil the essential subjectivity of attractiveness. The subjectivity emerges more evidently in the present scheme, since the reduction of the number of face space dimensions allows to avoid the undersampling occurring in experiments in which the subjects are asked to choose or rate natural faces.

We believe that the generality and reliability of the present approach could have a strong impact on future studies about beauty and pleasantness in different domains.

Possible completions of the present work are: an assessment of the robustness of principal components; an analysis of the intra-subject correlation matrix of facial coordinates; a variant of the analysis of correlations in an experiment with real facial images (whose landmarks could be automatically identified with deep learning techniques^46^); an unsupervised inference analysis of the database (already being carried on in our group) within the framework of the Maximum Entropy method.

## Methods

### Face space

Our experimental design is based on the parametrisation of the face in a 10-dimensional face-space defined by *D* = 11 vertical and horizontal *inter-landmark distances*, ${\bf{d}}={({d}_{i})}_{i=0}^{D}$ between standard facial landmarks (see Fig. 1A). The inter-landmark distances are subject to a constraint ${\rm{h}}={\sum }_{i=1}^{4}{d}_{i}=1$, reflecting the intrinsic scale invariance of the problem, in such a way that all distances *d*_*i*_ are in units of the total facial length (i.e., they represent *proportions with respect to the facial length*, rather than absolute distances). As vector of facial coordinates **f**, we have considered both the 11 distances *f*_*i*_ = *d*_*i*_ themselves or, alternatively, the non-redundant (and unconstrained) subset of *D* = 10 *Cartesian landmark coordinates* of a set of landmarks ${\overrightarrow{\ell }}_{\alpha }=({x}_{\alpha },{y}_{\alpha })$ (with *α* = 1, 3, 7, 9, 10, 12, 14, see Fig. 4 and Supplementary Sec. S13), that can be unambiguously retrieved from the set of inter-landmark distances. All the results presented in the article are qualitatively identical using the inter-landmark distances *d*_*i*_ or the landmark Cartesian coordinates ${\overrightarrow{\ell }}_{\alpha }$ as facial vectors.

### Separation of geometric and texture degrees of freedom

The face-space parametrisation is based, as previously mentioned, on the decoupling of *texture* (lightness, detailed, and skin textural) facial features, on the one hand, and *geometric* (landmark coordinates), on the other hand. The separation of these two kinds of degrees of freedom is a standard paradigm of face representation (see, for example^6,39,42^). It has been argued, in the light of the recently decoded neural coding for the facial identity in the primate brain, to be a naturally efficient parametrisation of the face^42^, outperforming other techniques in which texture and landmark-based are not separated, as the description in terms of *eigenfaces*.

### Image deformation

Given a reference portrait (see Fig. 1B) and a vector of facial distances **d**_1_, we create, by means of image (similarity transformation) deformation algorithms^47^, a realistic facial image based on the reference portrait, deformed in such a way that the inter-landmark distances defined in Fig. 1A assume the desired values, **d** = **d**_1_. Given the reference portrait image ${ {\mathcal I} }_{0}$, the position of its corresponding landmarks ${\overrightarrow{\ell }}_{\mathrm{0,}\alpha }$, and the vector **d**, we calculate the Cartesian coordinates ${\overrightarrow{\ell }}_{\mathrm{1,}\alpha }$ of the new set of landmarks, completely defined by **d**. The image deformation algorithm then generates a new facial image ${ {\mathcal I} }_{1}$ with a point-dependent parameter linear transformation, such that the pixels occupying the landmark positions ${\overrightarrow{\ell }}_{\mathrm{0,}\alpha }$ in the original image are mapped into the new positions ${\overrightarrow{\ell }}_{\mathrm{1,}\alpha }$, and the rest of the pixels of the original image are mapped in order to produce a resulting image as realistic as possible. We have observed that, in order to produce realistic results, the linear transformation should be in the *similarity class*^47^, beyond *affine* transformations. The deformed image is actually not created by mapping every pixel of the original image, but only the corners of a sub-grid; the sub-images inside each sub-grid are then warped to a polygon defined by the mapped corners of the grid, through affine transformations. The size of the sub-grid is taken to $\lesssim 15$ pixels. Both the reference portrait and the deformed images are roughly 300 × 400 pixels for RP1-2.

### Genetic algorithm of face-space exploration

The genetic algorithm is based on a sequence of pairwise subject’s choices among two facial images that are adaptively proposed to the subject, learned from his/her previous choices. An initial *population* of *N* vectors of randomised facial coordinates, **f**^(*s*,*n*)^(0), evolve by means of genetic mutation and recombination, subject to the *selection* exerted by the experimental volunteer. At the *t*-th generation, the *N* vectors of the population generate an offspring of *N* individuals, by mutation and recombination according to the *differential evolution algorithm* (see Supplementary Sec. S3). The offspring is generated from the facial vectors only, independently of the reference portrait. The subject plays then the role of the evolutive pressure in the algorithm dynamics, selecting (*N* times) one among *two facial images*: one made from a vector of the population (and a reference portrait), and one made from its offspring. The *t* + 1-th generation of vectors is then taken as the *N* vectors selected by the subject at the *t*-th generation. After a certain number, *T*, of generations, the population of facial vectors eventually reaches a regime in which the population of vectors do not change too much from one generation to the next. The *T*-th population of facial vectors is taken as the population of vectors sculpted by the subject, and constitutes the outcome of experiments E1-3.

This approach differs from previous approaches to facial attractiveness based on genetic algorithms^48,49^ in what: it allows a subject to select *in real time* a *realistic* facial image; in terms of geometric quantities only; with *fixed texture degrees of freedom*; finally, *avoiding the use of numerical ratings*, since the subject performs a sequence of left/right choices rather than assigning ratings to the images.

Populations of facial vectors sculpted by different subjects tend to be more far apart than populations sculpted by the same subject (see Sec. Results). Remarkably, the real difference between different subjects’ attractors is even larger, since it is unavoidably underestimated in virtue of the finiteness of the experimental method. Indeed, two standard deviations with different origins contribute to the self-consistency distance *μ*_sc_ (see Fig. 3). One is the intrinsic, cognitive ambiguity of the subject’s criterion; the other is the uncertainty brought by the genetic algorithm stochasticity (sec. Supplementary Sec. S3), whose origin is the discreteness of the proposed mutations and the consequent stochastic bias in the face space exploration. In genetic experiments with parameters in what we call in the *slow search regime* (mainly larger *N* and number of generations, *T*), the algorithmic uncertainty decreases, and *μ*_sc_ is expected to decrease consequently. This is the general expected behaviour of the differential evolution algorithm. We have also verified this fact experimentally: the distances among populations sculpted by a single subject significantly decrease for increasing values of *N* = 10, 20, 28. As a consequence, variants of the present experiment with *slower* genetic algorithm parameters would more finely *resolve* different subject’s facial ideals, leading to a larger gap between inter-subject and self-consistency distances, at the cost of a larger number of subject’s choices and experimental time.

### Details of the experiments

Experiments E1, E2, E3 were performed by a pool of *S* = 95 volunteers (54 female, 39 male, of age average and standard deviation: 26(12)), mainly students, researchers and professors of the University “La Sapienza”. Experiment E2 was performed under identical conditions of E1. A subset of *S*_sc_ = 6 participants to E1 (3 females, 3 males, of age average and standard deviation: 33(15)), were asked to perform 5 further instances of the experiment E1, in five different days, using, as in E1, the reference portrait RP1. The genetic algorithm parameters used are (see Supplementary Sec. S3): *N* = 28, *T* = 10, *μ* = 0.15, *ρ* = 1. Each subject performed a number of *NT* = 280 choices among couples of facial images. These are 400 × 300 pixel, B/W images in an 1024 × 768 resolution monitor. The reference portraits RP1-2 have been taken from the *Chicago face database*^50^. Each experiment lasted roughly 25 minutes on average (see the histogram of time intervals among successive left-right choices in figure Supplementary Sec. S7). The subjects were asked to look away and relax for some second each *N* = 28 choices. All methods in experiments E1-3 were carried out in accordance with relevant guidelines and regulations. The experimental protocols used have been approved by the General Data Protection Regulation (EU) 2016/679. Informed consent was obtained from all subjects. No subjects under 18 participated in the experiment.

## Supplementary information

SUPPLEMENTARY INFORMATION Subjectivity and complexity of facial attractiveness

## Data Availability

The data and the codes devoted to the data analysis are available by request to the corresponding author.

## References

[1] Bzdok D (2011). ALE meta-analysis on facial judgments of trustworthiness and attractiveness. Brain Structure and Function.

[2] Hahn Amanda C., Perrett David I. (2014). Neural and behavioral responses to attractiveness in adult and infant faces. Neuroscience & Biobehavioral Reviews.

[3] Laurentini A, Bottino A (2014). Computer analysis of face beauty: A survey. Computer Vision and Image Understanding.

[4] Little AC (2014). Facial attractiveness. Wiley Interdisciplinary Reviews: Cognitive Science.

[5] Thornhill R, Gangestad SW (1999). Facial attractiveness. Trends in Cognitive Sciences.

[6] Walker M, Vetter T (2016). Changing the personality of a face: Perceived Big Two and Big Five personality factors modeled in real photographs. Journal of personality and social psychology.

[7] Little A. C., Jones B. C., DeBruine L. M. (2011). Facial attractiveness: evolutionary based research. Philosophical Transactions of the Royal Society B: Biological Sciences.

[8] Leopold DA, Rhodes G (2010). A Comparative View of Face Perception. Journal of comparative psychology (Washington, D.C.: 1983).

[9] Maret SM, Harling CA (1985). Cross-cultural perceptions of physical attractiveness: Ratings of photographs of whites by cruzans and americans. Perceptual and Motor Skills.

[10] Langlois JH (2000). Maxims or myths of beauty? a meta-analytic and theoretical review. Psychological bulletin.

[11] Cunningham Michael R., Roberts Alan R., Barbee Anita P., Druen Perri B., et al (1995). "Their ideas of beauty are, on the whole, the same as ours": Consistency and variability in the cross-cultural perception of female physical attractiveness. Journal of Personality and Social Psychology.

[12] Bernstein IH, Lin T-D, McClellan P (1982). Cross- vs. within-racial judgments of attractiveness. Perception & Psychophysics.

[13] Thakerar JN, Iwawaki S (1979). Cross-cultural comparisons in interpersonal attraction of females toward males. The Journal of Social Psychology.

[14] Perrett DI, May KA, Yoshikawa S (1994). Facial shape and judgements of female attractiveness. Nature.

[15] Perrett D. I., Lee K. J., Penton-Voak I., Rowland D., Yoshikawa S., Burt D. M., Henzi S. P., Castles D. L., Akamatsu S. (1998). Effects of sexual dimorphism on facial attractiveness. Nature.

[16] Alley Thomas R., Cunningham Michael R. (1991). Article Commentary: Averaged Faces Are Attractive, but Very Attractive Faces Are Not Average. Psychological Science.

[17] Valenzano DR, Mennucci A, Tartarelli G, Cellerino A (2006). Shape analysis of female facial attractiveness. Vision Research.

[18] Cunningham MR, Barbee AP, Pike CL (1990). What do women want? facialmetric assessment of multiple motives in the perception of male facial physical attractiveness. Journal of personality and social psychology.

[19] Edler RJ (2001). Background considerations to facial aesthetics. Journal of Orthodontics.

[20] Valentine T, Lewis MB, Hills PJ (2016). Face-space: A unifying concept in face recognition research. The Quarterly Journal of Experimental Psychology.

[21] Hill H (2011). How different is different? criterion and sensitivity in face-space. Frontiers in Psychology.

[22] Joy, K. L. & Primeaux, D. A Comparison of Two Contributive Analysis Methods Applied to an ANN Modeling Facial Attractiveness. In *Fourth International Conference on Software Engineering Research, Management and Applications (SERA’06)*, 82–86, 10.1109/SERA.2006.2 (2006).

[23] Schmid K, Marx D, Samal A (2008). Computation of a face attractiveness index based on neoclassical canons, symmetry, and golden ratios. Pattern Recognition.

[24] Pallett PM, Link S, Lee K (2010). New golden ratios for facial beauty. Vision Research.

[25] Fan Jintu, Chau K.P., Wan Xianfu, Zhai Lili, Lau Ethan (2012). Prediction of facial attractiveness from facial proportions. Pattern Recognition.

[26] Shen, H. *et al*. Brain responses to facial attractiveness induced by facial proportions: evidence from an fMRI study. *Scientific Reports* **6**, 10.1038/srep35905 (2016). 10.1038/srep35905 PMC5078804 27779211

[27] Gray D, Yu K, Xu W, Gong Y (2010). Predicting facial beauty without landmarks. Computer Vision–ECCV.

[28] Bottino Andrea, Laurentini Aldo (2012). The Intrinsic Dimensionality of Attractiveness: A Study in Face Profiles. Progress in Pattern Recognition, Image Analysis, Computer Vision, and Applications.

[29] Eisenthal Yael, Dror Gideon, Ruppin Eytan (2006). Facial Attractiveness: Beauty and the Machine. Neural Computation.

[30] Mu Y (2013). Computational facial attractiveness prediction by aesthetics-aware features. Neurocomputing.

[31] Gan J, Li L, Zhai Y, Liu Y (2014). Deep self-taught learning for facial beauty prediction. Neurocomputing.

[32] Bronstad PM, Langlois JH, Russell R (2008). Computational Models of Facial Attractiveness Judgments. Perception.

[33] Chen, Y., Mao, H. & Jin, L. A novel method for evaluating facial attractiveness. In *2010 International Conference on Audio, Language and Image Processing*, 1382–1386, 10.1109/ICALIP.2010.5685007 (2010).

[34] Xu, J. *et al*. Facial attractiveness prediction using psychologically inspired convolutional neural network (PI-CNN). In *2017 IEEE International Conference on Acoustics, Speech and Signal Processing (ICASSP)*, 1657–1661, 10.1109/ICASSP.2017.7952438 (2017).

[35] Galantucci LM, Gioia ED, Lavecchia F, Percoco G (2014). Is principal component analysis an effective tool to predict face attractiveness? a contribution based on real 3d faces of highly selected attractive women, scanned with stereophotogrammetry. Medical & Biological Engineering & Computing.

[36] Adolphs R, Nummenmaa L, Todorov A, Haxby JV (2016). Data-driven approaches in the investigation of social perception. Phil. Trans. R. Soc. B.

[37] Oosterhof NN, Todorov A (2008). The functional basis of face evaluation. Proceedings of the National Academy of Sciences.

[38] Todorov A, Oosterhof NN (2011). Modeling Social Perception of Faces [Social Sciences]. IEEE Signal Processing Magazine.

[39] Abir Yaniv, Sklar Asael Y., Dotsch Ron, Todorov Alexander, Hassin Ran R. (2017). The determinants of consciousness of human faces. Nature Human Behaviour.

[40] Farkas, L. G. *Anthropometry of the Head and Face*. (Raven Pr, 1994).

[41] Burton AM, Miller P, Bruce V, Hancock PJB, Henderson Z (2001). Human and automatic face recognition: a comparison across image formats. Vision Research.

[42] Chang L, Tsao DY (2017). The code for facial identity in the primate brain. Cell.

[43] Ibáñez-Berganza, M., Lancia, G. L., Amico, A., Monechi, B. & Loreto, V. Unsupervised inference approach to human facial preference. *(to be submitted)* (2019). 10.7717/peerj.10210 PMC7602690 33194411

[44] Rhodes Gillian (2006). The Evolutionary Psychology of Facial Beauty. Annual Review of Psychology.

[45] Little AC, Burt DM, Perrett DI (2006). What is good is beautiful: Face preference reflects desired personality. Personality and Individual Differences.

[46] Wang Nannan, Gao Xinbo, Tao Dacheng, Yang Heng, Li Xuelong (2018). Facial feature point detection: A comprehensive survey. Neurocomputing.

[47] Schaefer S, McPhail T, Warren J (2006). Image deformation using moving least squares. ACM Trans. Graph..

[48] Johnston VS, Franklin M (1993). Is beauty in the eye of the beholder?. Ethology and Sociobiology.

[49] Wong Brian J. F., Karimi Koohyar, Devcic Zlatko, McLaren Christine E., Chen Wen-Pin (2008). Evolving Attractive Faces Using Morphing Technology and a Genetic Algorithm: A New Approach to Determining Ideal Facial Aesthetics. The Laryngoscope.

[50] Ma DS, Correll J, Wittenbrink B (2015). The Chicago face database: A free stimulus set of faces and norming data. Behavior Research Methods.

