# Supplementary material for: Subjectivity and complexity of facial attractiveness
Source: Sci Rep. 2019 Jun 10;9:8364. doi: 10.1038/s41598-019-44655-9 (PMC6557895; doi:10.1038/s41598-019-44655-9)
Supplement: Supplementary file 1 — SUPPLEMENTARY INFORMATION Subjectivity and complexity of facial attractiveness [file 41598_2019_44655_MOESM1_ESM.pdf]

SUPPLEMENTARY INFORMATION
Subjectivity and complexity of facial attractiveness

Miguel Ibáñez-Berganza[1*], Ambra Amico[1], Vittorio Loreto[2,1,3]
[1] Sapienza University of Rome, Physics Department, Piazzale Aldo Moro 2, 00185 Rome, Italy
[2] Sony Computer Science Laboratories, Paris, 6, rue Amyot, 75005, Paris, France
[3] Complexity Science Hub, Josefstädter Strasse 39, A 1080 Vienna, Austria

Contents

1 Detailed description of the face-space 2
2 Beauty as extrema in a given coordinate space 4
3 Genetic Algorithm details 5
4 Calculation of observables and their errors 7
5 Assessment of the convergence of populations of vectors 10
6 Precision of the experiments 11
7 Response times 11
8 Distances among different partitions of the dataset 11
9 Averages and standard deviations of facial coordinates 13
10 Statistical distinguishability of partitions of the dataset 15
11 Pairwise correlations among facial coordinates 15
12 Image deformations along principal axes 16
13 Relevant angles 19
14 Application of the Maximum Entropy method 21
15 Higher order and spurious correlations 21
16 Bibliography 22

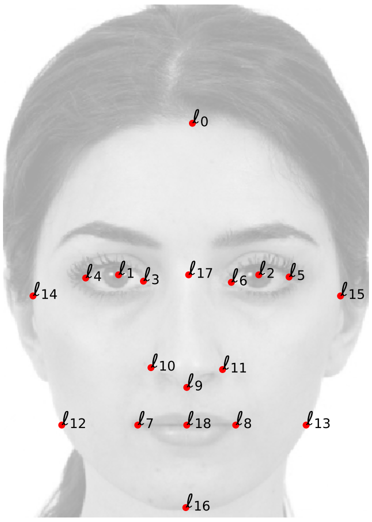

Figure 1: Facial landmarks used to define the face-space. The numbers correspond to the index α of various landmarks with Cartesian coordinates $\vec{\ell}_\alpha$.

1 Detailed description of the face-space

As explained in sections Results and Methods (main article), the face is parametrised as a vector $\mathbf{d} = (d_i)_{i=0}^{D}$ of 11 inter-landmark distances, shown in figure 1-A (main article). These are the vectors defining the "genoma" of the population members in the genetic algorithm. In the *selection* step of the genetic algorithm (see section 3), a facial image is generated from every vector $\mathbf{d}$ in the genetic population. This is done through the generation of an auxiliary 36-dimensional vector of Cartesian landmark coordinates, $\mathbf{L} = (\vec{\ell}_\alpha)_{\alpha=0}^{18}$ (with $\vec{\ell}_\alpha = (\mathsf{x}_\alpha, \mathsf{y}_\alpha)$), obtained from $\mathbf{d}$. The vector $\mathbf{L}$ is a list of the x, y coordinates (in pixels, the y growing downwards in the image) of the various landmarks evidenced in figure 1, labelled by α. The facial image corresponding to $\mathbf{d}$ is then generated, using the technique described in sec. Methods (main article), from the triplet $\{\mathcal{I}_0, \mathbf{L}_0, \mathbf{L}\}$, where $\mathcal{I}_0$ is the reference portrait image, $\mathbf{L}_0$ its Cartesian landmark coordinates, and $\mathbf{L}$ is the vector of Cartesian landmarks generated from the desired vector $\mathbf{d}$. The mapping $\mathbf{L} \leftrightarrow \mathbf{d}$ is one-to-one, given the set of Cartesian landmark coordinates of the reference portrait being used, $\mathbf{L}_0$. The information present in $\mathbf{L}_0$ is used to impose some constraints: the eye aspect ratio (the $\vec{\ell}_1$–$\vec{\ell}_3$ segment slope $(\mathsf{y}_3 - \mathsf{y}_1)/(\mathsf{x}_3 - \mathsf{x}_1)$) is constant and equal to that of $\mathbf{L}_0$, and the same is valid for $\vec{\ell}_1$–$\vec{\ell}_4$ (otherwise the pupil could become an ellipse); $\mathsf{y}_9 - \mathsf{y}_{10}$ is constant and equal to its value in $\mathbf{L}_0$. Moreover, the reference portrait determines the origin of the reference frame, the coordinate $\vec{\ell}_0$, which is fixed as well as $\vec{\ell}_{16}$. The information in $\mathbf{L}$ is highly redundant: by construction, the x coordinates of left/right landmarks are symmetric with respect to x_0 and share the y coordinate; the 12-th landmark is defined in such a way that its ordinate coincide with that of $7, 18, 8, 13$, and, analogously, $\mathsf{y}_1 = \mathsf{y}_{17} = \mathsf{y}_2$.

Let us now describe in detail the two parametrisations used to construct the face-space vectors $\mathbf{f}$.

i	type (h/v)	definition	name
0	v	$\mathsf{y}_{14} - \mathsf{y}_0$	zygomatic bone ordinate
1	v	$\mathsf{y}_{17} - \mathsf{y}_0$	eye-forehead distance
2	v	$\mathsf{y}_9 - \mathsf{y}_{17}$	nose length
3	v	$\mathsf{y}_{18} - \mathsf{y}_9$	nose-mouth distance
4	v	$\mathsf{y}_{16} - \mathsf{y}_{18}$	chin-mouth distance
5	h	$\mathsf{x}_{15} - \mathsf{x}_{14}$	face width
6	h	$\mathsf{x}_2 - \mathsf{x}_1$	inter-eye distance
7	h	$\mathsf{x}_8 - \mathsf{x}_7$	mouth width
8	h	$\mathsf{x}_3 - \mathsf{x}_4$	eye width
9	h	$\mathsf{x}_{11} - \mathsf{x}_{10}$	nose width
10	h	$\mathsf{x}_{13} - \mathsf{x}_{12}$	jaw width

Table 1: Definition of the *inter-landmark distance* facial coordinates f_i in terms of the horizontal/vertical (h/v) distances among landmark coordinates $\vec{\ell}_\alpha$ (see figure 1).

i	$\alpha(i)$	$\mathsf{c}(i) = \mathsf{x}, \mathsf{y}$	name
0	1	x	left pupil abscissa
1	3	x	internal left eye limit abscissa
2	7	x	left mouth limit abscissa
3	10	x	left outermost nose limit abscissa
4	14	x	zygomatic bone abscissa
5	12	x	left jaw limit (at the mouth's height) abscissa
6	1	y	left pupil ordinate
7	7	y	left mouth ordinate
8	9	y	nose endpoint ordinate
9	14	y	zygomatic bone ordinate

Table 2: Definition of the *Cartesian landmark* facial coordinates $f_i = \mathsf{c}(i)_{\alpha(i)}$ in terms of the landmark coordinates $\vec{\ell}_\alpha = (\mathsf{x}_\alpha, \mathsf{y}_\alpha)$ (see figure 1). For each coordinate i we specify $\alpha(i)$ and $\mathsf{c}(i) = \mathsf{x}$ or y.

- *Inter-landmark distances.* The facial vector components are taken as the 11 distances in $\mathbf{d}$

$$f_i = d_i \tag{1}$$

  The names of the 11 resulting facial coordinates are shown in table 1.

- *Reduced set of Cartesian landmark coordinates.* The facial vector components are taken as a vector of non-redundant, *reduced* set of $D = 10$ Cartesian landmark coordinates, or D non-redundant components of the vector $\mathbf{L}$. In other words:

$$f_i = \mathsf{c}(i)_{\alpha(i)}, \tag{2}$$

  with $\alpha(i) = 1, 3, 7, 9, 10, 12, 14$ and $\mathsf{c}(i) \in \{\mathsf{x}, \mathsf{y}\}$, specified in table 2.

Both sets of coordinates such defined are equally dimensional: the inter-landmark distances $\mathbf{d}$ have one more degree of freedom, but are subject to the constraint $\mathsf{h} = \sum_{i=1}^{4} d_i = 1$, so that the dimensionality of both sets is 10. As previously stated, the mapping among both vectors is one-to-one: they contain the same information, and are such that there is no redundant information in the reduced $\mathbf{L}$ (given the reference portrait vector).

All the observables that we have calculated in the data analysis (see section 4) can be computed in terms of $\mathbf{d}$'s or in terms of reduced $\mathbf{L}$'s. All the results presented in this article are qualitatively identical using either face-space parametrisation. Some results are clearer in terms of $\mathbf{L}$'s, due to the presence of the constraint $\mathsf{h} = 1$ to which the $\mathbf{d}$ vectors are subject. This is the case of the correlation matrix, that we have decided to show in figure 9 in terms of reduced $\mathbf{L}$'s (see below). The correlation matrix in terms of inter-landmark distances will be analysed in-depth in [1].

2 Beauty as extrema in a given coordinate space

As explained in the main text, our experimental setup allows a subject to sculpt a population of facial vectors, considered as an empirical sample of his/her attractor, or preferred region in face-space. The efficient characterisation of the attractor from a finite set of binary choices by the subject is an *inference* problem, that we tackle as an optimization problem, solved with the use of a genetic algorithm. This strategy is justified and motivated through the following assumptions. Given an experimental subject and a parametrisation of the human face in a real-valued vector, we assume that there exists a region of the face-space that represents the subject's preference, in the sense that he/she would statistically tend to prefer images in that region rather than those farther from it, and that this region can be probabilistically characterised within some accuracy. We postulate the existence of a subject-dependent probability distribution in the face-space, $\mathcal{L}_{\mathrm{g}}$, such that $\mathcal{L}_{\mathrm{g}}(\mathbf{f})/\mathcal{L}_{\mathrm{g}}(\mathbf{f}')$ represents the relative probability of the subject to express his/her preference for the facial image whose coordinates are $\mathbf{f}$ (so that a flat function represents a completely indifferent or unpredictable subject) [A convex function, locally flat around a set of points $\mathbf{f}^*$, $\nabla\mathcal{L}|_{\mathbf{f}^*} = \mathbf{0}$, $H = \det \mathrm{Hess}[\mathcal{L}](\mathbf{f}^*) < 0$ would represent a subject which tends to refuse local variations away from such set, with a probability depending on the modulus of H .] If the subject could modify the coordinates of an image following his/her personal taste, he/she would tend to finally choose the face-space region corresponding to the relative extrema of the function $\mathcal{L}_{\mathrm{g}}$ (if the facial image details, not parametrised by $\mathbf{f}$, are unchanged and given by the reference portrait). Of course, in real experiments, the function $\mathcal{L}_{\mathrm{g}}$ can only be inferred with uncertainty (induced, at least, by the subject's uncertainty), leading to an inferred function, $\mathcal{L}$. The present experimental scheme provides a finite set of representative vectors sampled with experimental uncertainty from $\mathcal{L}_{\mathrm{g}}$; hence, a function $\mathcal{L}$ may be inferred from such representative vectors, using the Maximum Entropy method [1]. The inference quality depends on both the extent to which the set of populations is representative of the subject's attractor (a first-step, experimental inference), and on the inference procedure of the set of populations

(the second-step inference, leading to $\mathcal{L}$). Under these working hypotheses, one is allowed to treat the inference of the single-individual attractor as an optimisation problem of a multi-valued function that, however, cannot be evaluated numerically. We now explain how this is done in our experimental scheme.

3 Genetic Algorithm details

Let us specify the details of the genetic algorithm for face-space exploration. Each instance of the experiment is defined by a set of algorithm parameters, $\mathcal{P} = \{N, T, \rho, \mu\}$. The algorithm defines a stochastic, discrete time dynamics (the time index t) of the population of facial vectors $\mathbf{f}^{(s,n)}(t)$, $n = 1, \ldots, N$, coupled to the dynamics of an abstract subject s, which performs binary choices among couples of vectors according to some stochastic rule. Even if the subject's binary choices were deterministic, the dynamics is intrinsically stochastic in the initial condition and in the sequence of random numbers. **(1) Initialization**. At $t = 0$, the initial vectors $\mathbf{f}^{(s,n)}(0) = \boldsymbol{\xi}_{(s,n)} + \mathbf{f}_0$ are taken as N facial vectors whose coordinates are random, uncorrelated, zero-averaged fluctuations around a given (common to all subjects) facial vector $\mathbf{f}_0$. **(2) Recombination and mutation**. For each of the N facial vectors in the population, $\mathbf{f}$, a *child*, or potential offspring vector $\mathbf{v}$, is generated, according to a rule specified in the next paragraph (based on recombination and stochastic mutation of the existing vectors in the population). **(3) Selection**. For each of the N couples of vectors of the original and of the offspring population, $\mathbf{f}, \mathbf{v}$, a pair of facial images $\mathcal{I}(\mathbf{f})$, $\mathcal{I}(\mathbf{v})$ is generated (with the image deformation algorithms described in sec. Methods (main article). Afterwards, the subject chooses among the two images (the one corresponding to a vector belonging to the current generation, and the one corresponding to *its child*), for each of the N pairs. The N chosen facial vectors (of which some are offspring and some are parents in the t-th generation) will form the successive generation; **(4)**. One now goes recursively to **(2)** and $t+=1$ until $t = T$.

Differential Evolution Algorithm. The rules in step **(2)** are given by a particular genetic algorithm called Differential Evolution Algorithm [2, 3]. It has been chosen due to its suitability to find multiple extrema and to the fact that it does not require the numerical evaluation of the function to be maximised, say $\mathcal{L}$, but only the boolean inequality $\mathcal{L}[\mathbf{f}\,] < \mathcal{L}[\mathbf{v}]$. In our experiment, the evaluation of this inequality corresponds to the choice of the subject between two images $\mathcal{I}(\mathbf{f})$, $\mathcal{I}(\mathbf{v})$. Given the population at time t, the *son*, $\mathbf{v}^{(j)}$, of the $j-$th vector of the population $\mathbf{f}^{(j)}(t)$, is generated from this vector and from two different *parents*, $\mathbf{f}^{(j_1)}$, $\mathbf{f}^{(j_2)}$, with $1 \leq j_1 \neq j_2 \neq j \leq N$, randomly selected. The mutation and recombination steps are, $\forall i = 1, \ldots, D$:

$$v_i^{(j)} = \begin{cases} f_i^{(j)}(t) + \mu\,(f_i^{(j_1)}(t) - f_i^{(j_2)}(t)) & \text{with prob. } \rho \\ f_i^{(j)}(t) & \text{with prob. } 1-\rho \end{cases}$$

The selection and generation update steps can be written as:

$$\mathbf{f}^{(j)}(t+1) = \begin{cases} \mathbf{v}^{(j)} & \text{if } \mathcal{L}[\mathbf{f}^{(j)}(t)] < \mathcal{L}[\mathbf{v}^{(j)}] \\ \mathbf{f}^{(j)}(t) & \text{otherwise} \end{cases}$$

Note that the algorithm acts on every i-th single coordinate independently. ρ is called the *crossover probability* and μ the *mutation factor*, quantifying the amount of stochastic mutation in the genetic evolution. In our experiment, the evaluation of the inequality (3) corresponds to the choice between the corresponding facial images, $\mathcal{I}[\mathbf{v}^{(j)}]$ and $\mathcal{I}[\mathbf{f}^{(j)}(t)]$, by the subject. He/she chooses among the images corresponding to the first parent's vector and its child; the selected vector survives and becomes part of the successive generation.

The FACEXPLORE software operates, in this way, in a regime defined by four parameters, ρ, μ, N and T (and by other algorithm details, such as the way in which the initial population of vectors is initialised, the reference portrait, the constraints imposed to the facial vectors at each generation, or the size of the sub-grid to be warped in the image deformation algorithm, see sec. Methods (main article)). Especially for large values of ρ, and for small values of μ, N and T, the results of single realisations of the experiment depend on the sequence of random numbers and on the random initial condition used in the particular realisation. This is a general characteristic of the genetic algorithm, arising also in the optimisation of deterministic functions, not a specific characteristic of the FACEXPLORE software. For lower values of N, the algorithm does not perform an exhaustive local search in the parameter space at each generation. The offspring generation is biased by its finiteness, a fact that conditions the experimental course and, consequently, the outcome. Depending on the parameters $\mathcal{P}$, the algorithm stochasticity could become large enough to hinder the differences among different subjects' choices. The parameters can also be such that the stochasticity is moderate, in the sense that they allow to *resolve* the single subject peculiarities, whose existence has been demonstrated in the main article. Thus, for different values of the parameters $\mathcal{P}$, we can define two main situations:

- For large ρ and small μ, N and T (*fast-search regime*): the algorithm "converges fast" (in the sense of Fig. 2 (main article)), but the resulting final populations vary significantly when the subject repeats the experiment.
- For sufficiently small ρ and sufficiently large μ, T and, above all, N (what we call the *slow-search regime*): the experiment requires more choices to "converge", but the resulting population will respond more to the subject's criteria and less to the randomness, i. e., to the particular realization of the experiment.

Ideally, at the end of the process, the population of vectors reaches a pseudo-stationary regime which is stable against local fluctuations, i. e. that the user does not want to change. For experiments deep in the slow-search regime, the *average* differences between the outcomes of different realizations of a single-subject experiment, $\mu_{\rm sc}$, should no longer depend on the algorithm parameters ($\mu_{\rm sc}$ would not decrease using *slower* parameters $\mathcal{P}$). In this case, the only experimental uncertainty would be the subject's uncertainty. In practice, such a deep slow-search regime would require a large number of choices. The algorithm parameters must satisfy a compromise between the desired accuracy and the time required by the subject to perform the experiment.

A crucial point is how the initial population of vectors is selected. If the standard deviations of $\boldsymbol{\xi}$ are large enough, and for sufficiently large N, the

initial population covers a broad region of the face-space (actually, the first-generation facial images often result grotesque and misshapen). Together with a large value of N, T, μ and $1-\rho$, it is expected that, again, the initial condition $\boldsymbol{\xi}_{(s,n)}$ and $\mathbf{f}_0$, do not influence the outcome of the experiment.

The differential evolution algorithm described above generates an offspring population of vectors by addition of a component-wise fluctuation (proportional to the mutation constant μ, see equation 3), whose average amplitude is proportional to the component-wise distance between two members of the parent population. For larger values of t the vectors in a population tend to be confined nearer to the extrema or saddle points of the function $\mathcal{L}$; their average distance tend to decrease and, consequently, also the mutation fluctuations. This feedback loop is such that the distance among population vectors unavoidably tend to decrease, especially in the fast-search regime. The velocity with which the distance among intra-population vectors decreases is an estimation of the steepness of the function $\mathcal{L}$ to be maximised, around its maxima (or of the subject's criterion definiteness, see 2).

Alternative way of assessing the subject self-consistency. In experiment E2 we have estimated the consistency of different volunteers' criteria, comparing how close are the populations of vectors sculpted by a given subject in the final generation, $\{\mathbf{f}^{(s,n)}(T)\}_n$, of different realisations of the experiment. An alternative way of "sampling" the relevant face-space region of a subject (hence comparing different subjects' relevant regions) could be that of performing a longer experiment in which a proper stationary state is reached, such that the populations in the latest generations of the genetic experiment are statistically indistinguishable. If it exists, the stationary state (under a *stationary* dynamical rule), should not depend on the initial condition and on the sequence of numbers. This would require, of course, to modify the algorithm (that otherwise necessarily tends to produce closer and closer generations) with the addition of a fluctuation term that is constant in time:

$$v_i^{(j)} = f_i^{(j)}(t) + \mu(f_i^{(j_1)}(t) - f_i^{(j_2)}(t)) + \chi_i \qquad \text{with prob. } \rho \tag{3}$$
$$v_i^{(j)} = f_i^{(j)}(t) \qquad \text{with prob. } 1-\rho \tag{4}$$

being $\boldsymbol{\chi}(t)$ a D-vector of uncorrelated random numbers (in t and in their component) with null average and small (smaller than $\mu_{\rm sc}$), fixed standard deviation. We propose this variant of the algorithm as an alternative strategy for future experiments.

4 Calculation of observables and their errors

The observables that we have calculated to perform the data analysis are functions of the set of sculpted facial vectors, $\mathcal{S} = \{f_i^{(s,n)}\}_{s=1,n=1}^{S,N}$, defined in a general fashion, independent of the face-space parametrisation (restricted landmark Cartesian coordinates or inter-landmark distances). They are defined as the following.

- **Average of facial vectors.** $\langle f_i \rangle = (1/(SN)) \sum_{s,n} f_i^{(s,n)}$.

- **Standard deviation of facial vectors.** Crucially, the standard deviation of the single facial vector coordinate σ_i is not computed along both indices (s, n), $\mathrm{std}(\{f_i^{(s,n)}\}_{s,n})$, since the facial vectors within the population of a single individual are correlated. As a consequence, the naive standard deviation is an underestimation of the inter-subject standard deviation. The statistical error of the average, $\sigma_{\langle f_i \rangle}$ is calculated instead as a Bootstrap error, or the standard deviation of a series of $B \gg S$ averages of $\mathbf{f}$ over different subsamples $\mathcal{S}_b$ made by a random, identically uniformly distributed set of S subject indices. For each subject index s, only one population facial vector, n_s is considered:

$$\langle f_i \rangle_b = \frac{1}{S} \sum_{j=1}^{S} f_i^{(s_j(b), n_j(b))} \tag{5}$$

$$\sigma_{\langle f_i \rangle} = \mathrm{std}\left(\langle f_i \rangle_1, \ldots, \langle f_i \rangle_B\right) \tag{6}$$

where, for each b, $s_j(b)$, $n_j(b)$ are a set of S independently distributed (in b and in j) integer numbers in the intervals $[1 : S]$ and $[1 : N]$ respectively. In this way, the standard deviation of the average is computed over a set of averages where, in each one, S subjects are used (hence this error is proportional to $\sim S^{-1/2}$, as desired), and such that only uncorrelated (i.e., coming from different subjects) facial vectors are used in each average $\langle \cdot \rangle_b$. The inter-subject error of the single coordinate is then computed simply as:

$$\sigma_i = S^{-1/2}\, \sigma_{\langle f_i \rangle} \tag{7}$$

- **Standarised variables.** A set $\mathcal{Y}$ of standarised facial vector is constructed standarising each vector in $\mathcal{S}$:

$$y_j^{(s,n)} = (f_j^{(s,n)} - \langle f_j \rangle)/\sigma_j \tag{8}$$

- **Correlation matrix.** The correlation matrix of a standarised set of vector coordinates $\mathcal{G} = \{\mathbf{y}^{(s)}\}_{s=1}^{N}$ is computed as:

$$C_{ij}[\mathcal{G}] = \frac{1}{S} \sum_{s} y_i^{(s)} y_j^{(s)} \tag{9}$$

The matrix C used to compute the t- and p- values reported in table 4 and figure 9 is the average and standard deviation of this quantity over a *set of populations*. It is computed, again, with the Bootstrap method, in such a way that the sum in eq. 9 runs over different subject populations, and only one facial vector of each population is considered. The Bootstrap error corresponds to the standard deviation from subject to subject, proportional to $S^{-1/2}$. In general, the Bootstrap average and error of an observable $\mathcal{O}$ of the set of standarised vector populations $\mathcal{Y}$ is

$$\langle \mathcal{O} \rangle = \mathrm{average}\left(\langle \mathcal{O} \rangle_1, \ldots, \langle \mathcal{O} \rangle_B\right) \tag{10}$$

$$\sigma_{\mathcal{O}} = \mathrm{std}\left(\langle \mathcal{O} \rangle_1, \ldots, \langle \mathcal{O} \rangle_B\right) \tag{11}$$

where:

$$\langle \mathcal{O} \rangle_b = \frac{1}{S} \sum_{j=1}^{S} \mathcal{O}[\mathbf{y}^{(s_1(b),n_1(b))}, \ldots, \mathbf{y}^{(s_S(b),n_S(b))}] \tag{12}$$

Taking $\mathcal{O}[\mathcal{G}] = C[\mathcal{G}]$ in eq. 9, we obtain the average and standard deviation of the correlation matrix.

Since the averages have been subtracted in the standarised variables, the correlation matrix of a set of uncorrelated facial vectors vanishes within their Bootstrap errors, i.e., presents a large p-value, $p \sim 1/2$.

- **Principal components.** We define the set of principal components of the facial vectors, $\mathcal{Y}' = \{y'^{(s,n)}_i\}_{s=1,n=1}^{S,N}$, where a vector $\mathbf{y}'$ is the vector of the projections of the vector $\mathbf{y}$ along the various principal axes or eigenvectors of the correlation matrix, $\mathbf{y}' = E\mathbf{y}$ where E is the row-eigenvector matrix, $ECE^\dagger = \Lambda$ and $\Lambda = \text{diag}(\lambda_1, \ldots, \lambda_D)$, being λ_j the j-th eigenvalue of C.

- **Distances between sets of vectors.** Given two sets of vectors, $\mathcal{S}_1 = \{\mathbf{f}^{(s,n)}\}_{s=1,n=1}^{S_1,N}$, $\mathcal{S}_2 = \{\mathbf{g}^{(s,n)}\}_{s=1,n=1}^{S_2,N}$, the *inter-set pseudo-distance* is defined as:

$$\text{dist}(\mathcal{S}_1, \mathcal{S}_2) = \frac{1}{S_1 S_2} \sum_{s_1,s_2} \mathsf{D}(\mathbf{f}^{(s_1,\cdot)}, \mathbf{g}^{(s_1,\cdot)}) \tag{13}$$

where D is the (per coordinate) inter-population pseudo-distance, defined as:

$$\mathsf{D}(\mathbf{f}^{(s_1,\cdot)}, \mathbf{g}^{(s_2,\cdot)}) = \frac{1}{N^2} \sum_{n_1,n_2} \frac{1}{M} \mathsf{d}(\mathbf{f}^{(s_1,n_1)}, \mathbf{g}^{(s_2,n_2)}) \tag{14}$$

and where M is the dimension of the vectors $\mathbf{f}$, $\mathbf{g}$, and $\mathsf{d}(\mathbf{x_1}, \mathbf{x_2})$ is the *face-space metrics* or the distance between two single vectors in face-space. It can be defined in various ways (see [4, 5]):

1. **Euclidean-metrics.** As the Euclidean distance between the principal components of the vectors: $\mathsf{d}(\mathbf{f}_1, \mathbf{f}_2) = ||\mathbf{f}'_1 - \mathbf{f}'_2||$, where $|| \cdot ||$ is the Euclidean metrics in D dimensions, being $\mathbf{f}' = E\mathbf{f}$, and E the row-eigenvector of the last r eigenvectors of the correlation matrix (corresponding to non-standarised facial vectors). Taking $r = D$, it coincides with the Euclidean distance, $||\mathbf{f}_1 - \mathbf{f}_2||$.
2. **Euclidean-metrics with standarised vectors.** As the Euclidean distance between the principal components of the standarised vectors: $\mathsf{d}(\mathbf{y}_1, \mathbf{y}_2) = ||\mathbf{y}'_1 - \mathbf{y}'_2||$, being $\mathbf{y}' = E\mathbf{y}$, and E the row-eigenvector of the standarised correlation matrix.
3. **Mahalanobis-metrics.** As the Euclidean metrics between *standarised principal components* of the vectors, or: $\mathsf{d}(\mathbf{y}_1, \mathbf{y}_2) = ||\mathbf{y}''_1 - \mathbf{y}''_2||$, where $\mathbf{y}'' = \mathbf{y}'/\boldsymbol{\lambda}$, being $\mathbf{y}' = E\mathbf{y}$ (and $\boldsymbol{\lambda}$ the eigenvalues of the correlation matrix), the vector division meaning a component-wise division.

4. **Angle-metrics.** As the angle (in the D-dimensional face-space) subtended between two standarised principal components, or: $\mathsf{d}(\mathbf{y}_1, \mathbf{y}_2) = \arccos(\mathbf{y}_1'' \cdot \mathbf{y}_2'' / ||\mathbf{y}_1''||\,||\mathbf{y}_2''||)$.
5. **Byatt-Rhodes metrics.** As:

$$\mathsf{d}(\mathbf{y}_1, \mathbf{y}_2) = \frac{||\mathbf{y}_1'' - \mathbf{y}_2''||\,|\mathbf{y}_1''|\,|\mathbf{y}_2''|}{\mathbf{y}_1'' \cdot \mathbf{y}_2'' + \epsilon} \tag{15}$$

ϵ being a small regularising term.

All the results are quantitatively equivalent using instead the *min-inter-population* pseudo distance:

$$\mathsf{D}_{\min}(\mathbf{f}^{(s_1,\cdot)}, \mathbf{g}^{(s_2,\cdot)}) = \frac{1}{2}\frac{1}{N}\left\{\sum_{n_1}\min_{n_2} + \sum_{n_2}\min_{n_1}\right\}\left\{\frac{1}{M}\mathsf{d}(\mathbf{f}^{(s_1,n_1)}, \mathbf{g}^{(s_2,n_2)})\right\} \tag{16}$$

- **Reducing the number of principal components.** In the main text, we have also analysed the effect of reducing the number of principal components in the definition of the metrics. This is implemented as keeping in $\mathbf{y}'$ (and, consequently, in $\mathbf{y}''$) the principal components of $\mathbf{y}$ corresponding to the $r \le D$ highest eigenvalues of C only. If they are ordered in increasing order,: $\lambda_1 \le \lambda_2 \le \cdots \le \lambda_D$, this is $\mathbf{y}' = E\mathbf{y}$ being E the $r \times D$ matrix made by the last r row-eigenvectors.

- **Statistical errors of distances.** The statistical error associated to the metrics among sets of populations, equation 13, is the standard deviation of the argument in the sum across couples of different indices s_1, s_2. The statistical error associated to the inter-population pseudo-distance, equation 14, is the standard deviation of the argument in the sum across couples of different indices n_1, n_2. The latter error is lower than the former.

5 Assessment of the convergence of populations of vectors

The degree of coherence of the single subject's criterion in experiment E1 may be estimated through the degree of convergence of the population of vectors sculpted by the subject as a function of the generation index, t. In figure 2 (main article) we show the self-distance between the population of vectors sculpted by 10 randomly chosen subjects as a function of t. For a subject s, this quantity is (see 13):

$$d_{\text{conv}}^{(s)}(t) = \mathsf{D}(\mathbf{f}^{(s,\cdot)}(t), \mathbf{f}^{(s,\cdot)}(t)) \tag{17}$$

or the pseudo-distance between the population sculpted by the s-th subject at the t-th generation and itself. The figure errors have been calculated as explained in the precedent subsection.

Remarkably, different subjects exhibit different degrees of convergence. The reasons for such a diversity is an argument of possible cognitive interest in itself. A proposal for further work is to investigate the relation among the convergence velocity shown in Fig. 2 (main article), the subject's response times (see 7), and the self-consistency distance of each subject (see Fig. 3 (main article)).

In any case, after some generations, the populations of all the subjects in the sample result more self-distant (see Fig. 2 (main article)) than the populations resulting from a null model of genetic experiment, performed with the same parameters as in experiment E1 but in which the selection step (equation 3) is random. In Fig. 2 (main article), the error bars of the null test self-distance among populations refer to the standard deviation of the self-distance in the t-th generation across different realisations of the null model experiment.

6 Precision of the experiments

As previously explained, the vector of facial coordinates $\mathbf{f}$ contains the inter-landmark distance vector $\mathbf{d}$ or the reduced set of landmark Cartesian coordinates $\mathbf{L}$. In the first case, the coordinates represent distances in units of total facial length. Thus, they are not absolute distances, but proportions. In the second case, the coordinates correspond to pixels, divided by the reference portrait length in pixels. In both cases, they are floating point quantities, and the systematic error associated to the single vector is limited by the image resolution in pixels, $\gtrsim 400^{-1}$. This is roughly the precision with which we resolve the subject intra-population distance using the Euclidean metrics (per coordinate), i.e., the average distance along the single "physical" coordinate, see Fig. 4. The self-consistency distance in physical coordinates is, remarkably, barely twice than the image resolution. The average and standard deviation of self-consistency distances per coordinate using the Euclidean metrics is: 0.0045(9). This corresponds to a precision of 0.80(15)mm of the average female facial length. The intra-subject distances are estimated with an even higher precision (Fig. 4).

7 Response times

As explained in sec. 3, the **(3) selection** step of the genetic algorithm is implemented by the human subject, in our experimental scheme, as a choice among two facial images generated by the computer. In figure 2 we report the histogram of the ($S_1 NT$) elapsed time between consecutive left/right choices of every subject (with $NT = 280$ choices for each one) in experiment E1.

8 Distances among different partitions of the dataset

In figure 3 (main article) we report the self-consistency (among couples of populations sculpted by the same subject, for all the subjects in E2) and inter-subject (among couples of populations sculpted by different subjects in E1) distance histograms. The distances have been computed using the angle-metrics with

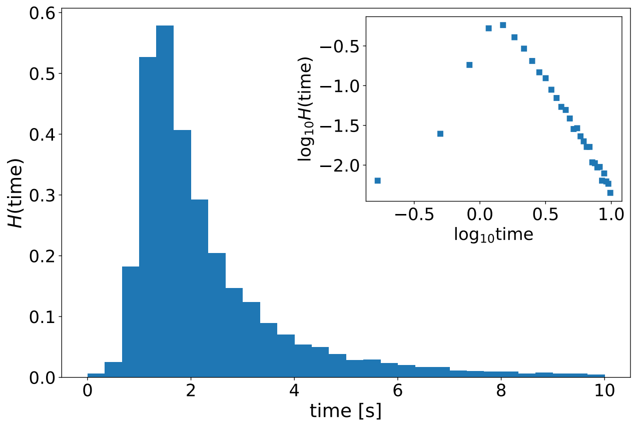

Figure 2: Histogram of delay time between consecutive left/right choices (between all the 280 choices of all subjects in E1). The most probable time is around 1.75s. Inset: the histogram in log-log scale.

$n_{\rm pc} = 7$ principal components. In figure 3 we present a comparison of the difference among histograms, $t_{12} = (\mu_1 - \mu_2)/(\sigma_1^2 + \sigma_2^2)^{1/2}$, using different values of $n_{\rm pc}$ in the face-space metrics definition. Such difference among histograms provides an estimation of the overlap among both distributions: the cumulative normal distribution of t_{12}, p_{12}, is actually the overlap probability of both histograms, if they are supposed to be normal distributions. The quantity t_{12} is, interestingly, a non-monotone function of $n_{\rm pc}$, the largest self-consistency/inter-subject distance is obtained with the angle-metrics using $n_{\rm pc} = 7$ principal components. For this metrics, the probability of two facial vectors sculpted by the same subject to be closer than two facial vectors sculpted by different subjects in E1 is p_{12} =0.79(1). This number coincides, within its statistical errors, with the empirical fraction of couples of inter-subject distances that are larger than a self-consistency distance. As mentioned before (see Sec. 3 for details), this probability is arguably underestimated, due to the finiteness of the experimental procedure.

Notice that σ_i^2 in the definition of t_{12} are the variances of the histograms, not the variances of the averages of the histograms, σ_i^2/N_i). These are used to compute the p-value of the histogram difference, corresponding to the Student's t-value $t = (\mu_1 - \mu_2)/(\sigma_1^2/N_1 + \sigma_2^2/N_2)^{1/2}$, practically equal to zero, $p < 10^{-30}$.

For completeness, in Fig. 4 we present the histograms corresponding to the intra-subject, inter-subject, self-consistent, inter-reference portrait and inter-subject gender sets of inter-population distances calculated with the Euclidean metrics in face-space (see below), i.e. the distance per coordinate using the D physical coordinates. Although the self-consistent and inter-subject histograms are more overlapping with respect to the angle-metrics histograms, they are still very obviously distinguishable ($p < 10^{-16}$, $p_{12} = 0.72(5)$, $t_{12} = 0.61(2)$).

Our experimental method succeeds to resolve the differences among single-subject preferred variations. This is possible thanks to the reduction of the

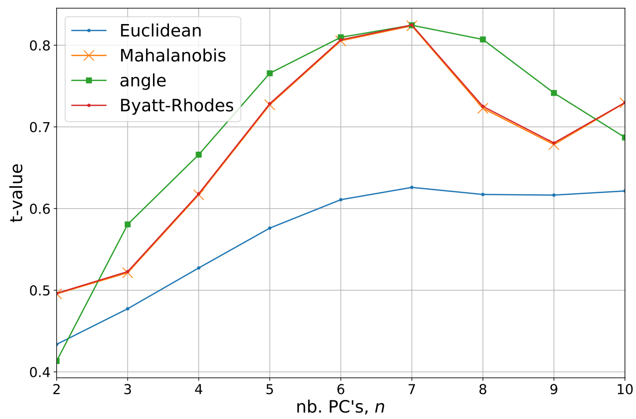

Figure 3: Histogram difference t_{12} among self-consistent and inter-subject population histograms of distances, versus the number of principal components considered in the definition of the face-space metrics. Different curves correspond to different kinds of face-space metrics.

face-space dimensionality. Such a reduction is implemented by considering geometrical degrees of freedom only, on the one hand, and, on the other hand, by keeping low the dimension of the (geometric) face-space.

The ideal number of dimensions is subject to the accuracy/complexity trade-off. A too low-dimensional face-space would not allow to detect the systematic inter-subject differences. As a limit case, think about a face space with a single inter-landmark distance, as the inter-eye distance: in this case, the subjects would clearly not result distinguishable. The differences among different subjects criteria are more complex, and involve, at least, several (linear combinations of) facial coordinates.

Conversely, a too high-dimensional face space would not allow a subject to sculpt a consistent (among several realisations) version of his/her attractor in the face-space in a reasonable time. In other words, the resulting set of sculpted faces obtained after a moderate number of choices (of pairwise choices in the software FACEXPLORE), would result less significant, or more dependent on the single realisation of the experiment, and less on the subjects criterion.

Such a trade-off is somehow reflected in the non-monotonic behaviour of t_{12} versus $n_{\rm pc}$.

9 Averages and standard deviations of facial coordinates

In figure 5 we show the results of the experimental averages, $\langle f_i \rangle$ and their standard deviations, σ_i, in terms of inter-landmark distances, $f_i = d_i$ (see sec. Methods (main article)). For all the coordinates, the standard deviations are much lower than the averages.

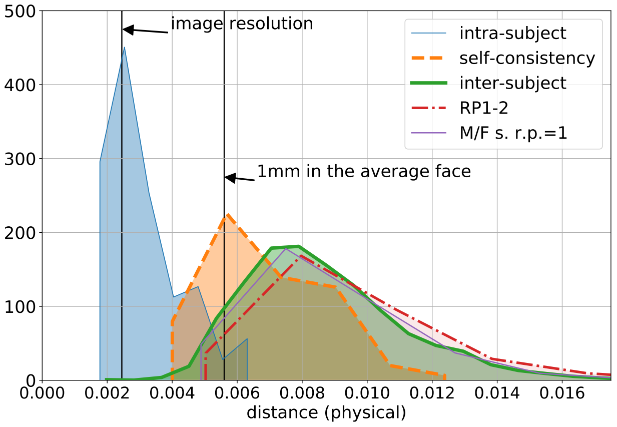

Figure 4: Histograms of intra-subject, self-consistent and inter-subject sets of inter-population distances (as shown in Fig. 3 (main article) for the angle-metrics), using the Euclidean-metrics in face-space with 11 coordinates (the "physical" distances). The arrows indicate the resolution of the image pixel (the distance among two pixels in units of the facial length, $\sim 400^{-1}$), and the scale corresponding to 1mm ($\ell_{\rm mm}^{-1}$, where $\ell_{\rm mm}$ is the female average facial length measured in mm).

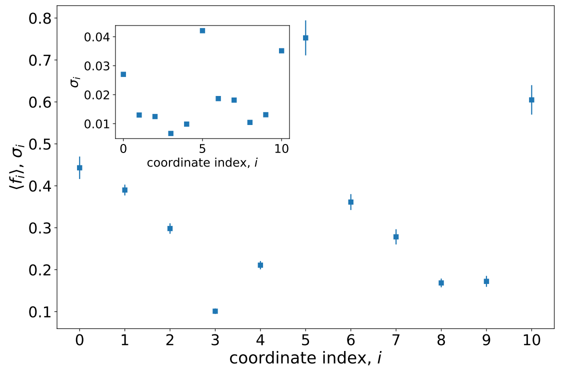

Figure 5: Averages and standard deviations (as error bars in the main figure, and in the inset) of the experimental facial coordinates, in terms of inter-landmark distances.

10 Statistical distinguishability of partitions of the dataset

We have performed single component (in physical, y_i or principal, y'_i coordinates) statistical tests between different sets of populations of facial vectors. Given two sets of populations, $\mathcal{S}_1 = \{\mathbf{f}^{(s,n)}\}_{s=1,n=1}^{S_1,N}$, $\mathcal{S}_2 = \{\mathbf{g}^{(s,n)}\}_{s=1,n=1}^{S_2,N}$, we consider the t-value, and the consequent p-value, of the differences among the averages of the y_i (or of the y'_i) coordinate in both sets of populations, $\langle f_i^{(s_1,\cdot)} - g_i^{(s_2,\cdot)}\rangle_{s_1,s_2}$. The average $\langle\cdots\rangle_{s_1,s_2}$ is performed again by bootstrapping, summing over all the couples (s_1,s_2) and over many ($B=500$) realisations in which different population indices $(n_1(s_1),n_2(s_2))$ are chosen for each tuple s_1,s_2. In this way, the error of this quantity is of order $\sim (S_1S_2)^{-1/2}$:

$$\langle f_i - g_i\rangle_b = \frac{1}{S_1S_2}\sum_{s_1,s_2=1}^{S_1,S_2} f_i^{(s_1,n_{1,b}(s_1))} - g_i^{(s_2,n_{2,b}(s_2))} \quad (18)$$

$$\langle f_i^{(s_1,\cdot)} - g_i^{(s_2,\cdot)}\rangle_{s_1,s_2} = \text{average}\left(\langle f_i-g_i\rangle_1,\cdots,\langle f_i-g_i\rangle_B\right) \quad (19)$$

$$\sigma(\langle f_i^{(s_1,\cdot)} - g_i^{(s_2,\cdot)}\rangle_{s_1,s_2}) = \text{std}\left(\langle f_i-g_i\rangle_1,\cdots,\langle f_i-g_i\rangle_B\right) \quad (20)$$

where, again, $n_{1,b}(s), n_{2,b}(s)$ are random, uncorrelated (in s, in b and in $1,2$) integers in $[1:N]$.

According to the reference portrait. In figure 6 we present the single component differences among the sets of vectors $\mathcal{S}_1$ and $\mathcal{S}_2$ described in sec. Results (main article), corresponding to the outcomes of experiments E1 and E3, respectively, in terms of inter-landmark distances as facial vectors, $y_i = d_i$. Only some facial coordinates are distinguishable (within our experimental errors, $\sim S^{-1/2}$) in both sets, specially d_i with $i = 3,5,8$. Qualitatively, the same result is found computing the differences of facial coordinates sculpted by the same subject with different portraits, $f_i^{(s,n_1)} - g_i^{(s,n_2)}$.

According to the subject's gender. In figure 7 we present the single component differences among the sets of vectors sculpted by female and male subjects in experiment E1, respectively, in terms of inter-landmark distances as facial vectors, $f_i = d_i$. Only some facial coordinates are distinguishable in both sets, specially d_i with $i = 1,2,8,9,10$ (eye height, nose height, eye width, nose width and zygomatic bone height). In figure 8 we show the same results but using the principal components, y'_i. Only some principal components ($i = 1,4,9$, see subsection 12 are distinguishable within the experimental errors. The principal component exhibiting largest variability, y'_{10}, is barely distinguishable in the female/male subject partition.

11 Pairwise correlations among facial coordinates

The list of the most strongly interacting couples of facial coordinates (the C_{ij} matrix elements with higher t_{ij}-value) is presented in terms of inter-landmark distances in table 3, and in terms of landmark Cartesian coordinates in table 4.

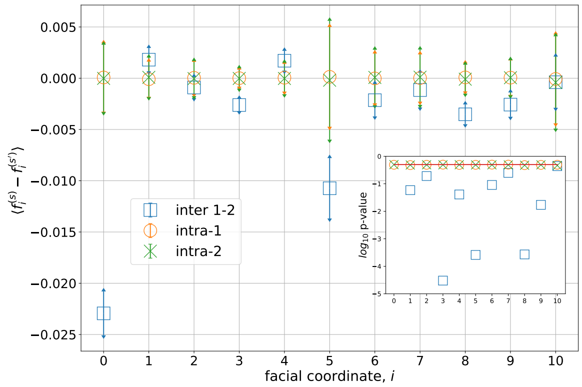

Figure 6: Impact of the reference portrait in different facial coordinates. Differences among facial coordinates $f_i^{(s,\cdot)} - f_i^{(s',\cdot)}$, with $f_i^{(s,\cdot)}$ and $f_i^{(s',\cdot)}$ belonging, respectively, to the set of populations of vectors sculpted in E1 and in E3, as a function of the coordinate index i (squares). Inter-landmark distances have been used as facial coordinates. Circles and crosses are the same quantity, but $f_i^{(s,\cdot)}$ and $f_i^{(s',\cdot)}$ belonging to a random partition of the E1 dataset (circles) and of the E3 dataset (crosses). The error-bars represent the Bootstrap standard deviation, $\sigma(\cdot)$, or the statistical fluctuations of the coordinate differences with respect to the number of subjects only. **Inset**: associated p-value (of the t-value, $(f_i^{(s,\cdot)} - f_i^{(s',\cdot)})/\sigma(\cdot)$). The $i = 2, 6, 7, 10$ coordinates result barely distinguishable or completely undistinguishable.

Fig. 9 presents the t_{ij} matrix elements corresponding to landmark Cartesian coordinates. The error has been calculated as specified in sec. 4.

12 Image deformations along principal axes

In figure 10 we report the facial images $\mathcal{I}[\mathbf{y}(i, \eta)]$ corresponding to the vectors:

$$\mathbf{y}(i, \eta) = \mathbf{0} + \eta\, \mathbf{e}^{(i)}$$

i.e., to a deformation of the average facial vector along the i-th principal axis, or the i-th eigenvector of C (from inter-landmark distances), in increasing order of eigenvalues, $\lambda_1 < \lambda_2 < \cdots < \lambda_{D-1}$ (the 0-th eigenvalue is null and correspond to the constraint $\mathsf{h} = 1$). Every row of figure 10 corresponds to a different eigenvector, while each column corresponds to a value of η in the set $-3q$, $-q/2$, 0, $q/2$, $3q$, $q = 0.075$ (the central column corresponding to the average facial vector). Higher rows, corresponding to lower associated eigenvalues λ_i, represent uncommon (say, unpleasant) deformations with respect to other axes (for equal η's), since their associated standard deviation, $\lambda_i^{1/2}$, is lower (c.f. figure 11). The $\mathbf{e}^{(1)}$ eigenvector, for instance, is a linear combination such that the

type	i, j	$\lvert C_{ij}\rvert$	$t_{ij} = C_{ij}/\sigma_{C_{ij}}$	p-value
hh	8,6	0.105	1.583	5.85e-02
hv	7,0	0.102	1.613	5.51e-02
hv	9,4	0.098	1.628	5.35e-02
hv	9,1	0.100	-1.677	4.85e-02
hv	10,0	0.104	1.722	4.42e-02
hv	10,2	0.111	-1.732	4.33e-02
hv	7,2	0.113	-1.779	3.93e-02
hv	9,0	0.104	1.807	3.70e-02
hv	8,3	0.112	1.963	2.63e-02
hh	9,7	0.101	1.985	2.51e-02
hh	9,5	0.104	2.054	2.14e-02
hh	9,8	0.103	2.118	1.85e-02
hh	6,5	0.121	2.119	1.84e-02
hv	10,3	0.109	2.133	1.78e-02
vv	3,1	0.093	-2.276	1.26e-02
hv	7,4	0.114	2.289	1.22e-02
hv	9,3	0.108	2.497	7.16e-03
hh	7,5	0.104	2.514	6.86e-03
hh	10,9	0.101	2.581	5.72e-03
vv	3,2	0.117	-2.643	4.84e-03
vv	4,2	0.109	-2.870	2.55e-03
hh	8,5	0.099	3.112	1.24e-03
hh	8,7	0.113	3.242	8.31e-04
hh	10,8	0.106	3.349	5.91e-04
hh	10,7	0.101	3.793	1.34e-04
vv	4,1	0.122	-3.872	1.02e-04
hh	10,5	0.121	3.878	9.94e-05
vv	2,1	0.116	-4.824	2.81e-06

Table 3: Relevant experimental correlations C_{ij} in terms of inter-landmark distances along with their corresponding t-value. v/h denotes the vertical/horizontal character of the involved coordinates d_i and d_j.

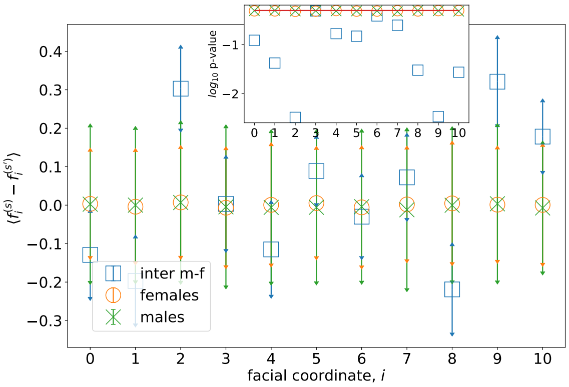

Figure 7: Impact of the subject's gender in different facial coordinates. Differences among facial coordinates $y_i^{(s,\cdot)} - y_i^{(s',\cdot)}$, with $y_i^{(s,\cdot)}$ and $y_i^{(s',\cdot)}$ belonging, respectively, to the set of populations of vectors sculpted by female and subjects in E1, respectively, as a function of the coordinate index i (squares). Inter-landmark distances have been used as facial coordinates. Circles and crosses are the same quantity, but $y_i^{(s,\cdot)}$ and $y_i^{(s',\cdot)}$ belonging to a random partition of the dataset of female (circles) and male subjects (crosses). Symbols, error-bars and inset are as in Fig. 6. The $i = 0, 3, 4, 6, 7$ coordinates result barely distinguishable or completely undistinguishable.

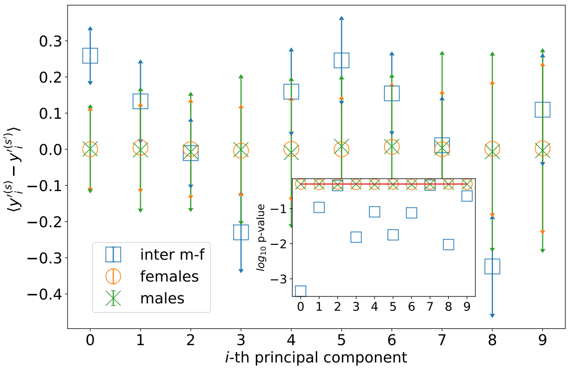

Figure 8: Impact of the subject's gender in different principal components. Differences among principal components of the facial vectors, ${y'}_i^{(s,\cdot)} - {y'}_i^{(s',\cdot)}$, with ${y'}_i^{(s,\cdot)}$ and ${y'}_i^{(s',\cdot)}$ belonging, respectively, to the set of populations of vectors sculpted by female and subjects in E1, respectively, as a function of the coordinate index i (squares). Symbols, error-bars and inset are as in figure 6. The $i = 3, 5, 7, 8, 10$ principal components result barely distinguishable or completely undistinguishable.

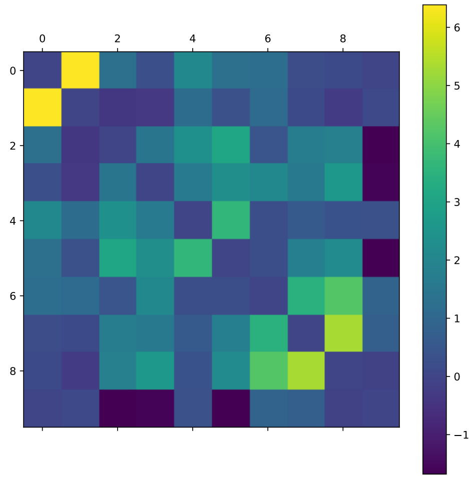

Figure 9: The t_{ij}-value corresponding to the correlation matrix among facial coordinates, $t_{ij} = C_{ij}/\sigma_{C_{ij}}$, in terms of Cartesian landmarks coordinates. The diagonal (equal to the unit vector $C_{ii} = 1$) has been set to zero for clarity.

face width increases while the jaw width decreases, or vice-versa, an uncommon, "forbidden" deformation according to the correlation matrix (see table 3), which indicates that these distances tend to increase or decrease together. Conversely, the lower rows, corresponding to high eigenvalues (larger than one, i.e., larger than the standard deviation of the single coordinates y_i), represent common deformations. The last eigenvector, $i = 10$, consists mainly in deformations in which the horizontal distances positively covary, by a roughly common, positive factor (see the $\mathbf{e}^{(10)}$ eigenvector components in figure 12). The eigenvectors $i = 1, 4, 9$ are the ones along which male and female subjects are more distinguishable.

13 Relevant angles

In sec. Results (main article) we have presented a geometric interpretation of *the sign* of the oblique correlation matrix elements in terms of some *relevant inter-landmark segment angles*, shown in figure 4 (main article). The sign of a given oblique matrix element (say, in terms of Cartesian landmark coordinates) $C_{ij} = \langle \delta \mathsf{y}_{\alpha(i)} \delta \mathsf{x}_{\alpha(j)} \rangle$ (with $\delta \mathsf{x}_\alpha = \mathsf{x}_\alpha - \langle \mathsf{x}_\alpha \rangle$, and the same for y) coincides with that of the slope of the average inter-landmark line, $\Delta_\mathsf{y}/\Delta_\mathsf{x}$, where $\vec{\Delta} = \langle \vec{\ell}_\alpha \rangle - \langle \vec{\ell}_\beta \rangle$. This is the only way in which the fluctuation of the α–β segment slope around its average value, $(\Delta_\mathsf{y} + \delta \mathsf{y}_\alpha)/(\Delta_\mathsf{x} + \delta \mathsf{x}_\beta) - \Delta_\mathsf{y}/\Delta_\mathsf{x}$ may vanish, at first order in the δ's.

This provides a clear interpretation of matrix C: the fluctuations generated by various subject's different aesthetic criteria are such that they tend to respect some *natural angles of the face, those defined by the inter-landmark segments evidenced in figure 4 (main article).* The figure shows the inter-landmark segments corresponding to the oblique correlations $\langle \mathsf{y}_\alpha \mathsf{x}_\beta \rangle$ *exhibiting higher t-*

landmarks involved	$\|C_{ij}\|$	t_{ij}	p-value
y_7x	0.109	1.675	4.90e-02
y_7x	0.109	1.685	4.80e-02
$y_{14}x_{10}$	0.105	-1.688	4.78e-02
$y_{14}x_{12}$	0.105	-1.722	4.46e-02
y_7x_7	0.135	1.723	4.45e-02
y_9x_7	0.132	1.748	4.23e-02
y_9x_{12}	0.115	2.092	1.99e-02
$x_{14}x_1$	0.132	2.098	1.97e-02
y_1x_{10}	0.109	2.124	1.85e-02
$x_{14}x_7$	0.117	2.164	1.68e-02
$x_{12}x_{10}$	0.110	2.196	1.56e-02
y_9x_{10}	0.111	2.700	4.28e-03
$x_{12}x_7$	0.113	3.003	1.82e-03
$x_{12}x_{14}$	0.142	3.245	8.79e-04
y_7y_1	0.129	3.513	3.77e-04
y_9y_1	0.124	4.234	3.22e-05
y_9y_7	0.170	5.018	1.70e-06
x_3x_1	0.136	6.495	4.03e-09

Table 4: Relevant experimental correlations C_{ij} in terms of landmark Cartesian coordinates, along with their corresponding t-value, $t_{ij} = C_{ij}/\sigma_{C_{ij}}$. The first column indicates $\mathsf{c(i)}_{\alpha(i)}\mathsf{c(j)}_{\alpha(j)}$, where $\alpha(i)$ is the landmark index involved and $\mathsf{c}(i) = \mathsf{x}$ or y.

value: perhaps representing the most relevant angles. One could be tempted, at this point, to attribute a *quantitative estimation of the relative importance* to each one of these segments, proportional to the C t-value or modulus. The following arguments suggest that this method is not the optimal way of assessing such relative relevance of various inter-landmark segments, and provide a further motivation to the application of the Maximum Entropy method to this problem.

A rigorous assessment of the relevance of various inter-landmark angles or slopes cannot be directly addressed from matrix C, since a slope, in the general case, is defined as a correlation among four landmark Cartesian (or inter-landmark distance) coordinates $((y_\alpha - y_\beta)/(x_\alpha - x_\beta))$. Furthermore, the empirical correlations are, in principle, an indirect manifestation of the *effective interactions* which cause them. The Maximum Entropy method allows to *infer* such effective interactions, from which we can more significantly assess the relative importance of various inter-landmark segments [1].

As can be seen in table 4 and figure 4 (main article), the sign of C_{ij} coincides with that of Δ_y/Δ_x for all the oblique C_{ij} elements, except $\langle y_9x_{10}\rangle$. This matrix element has a reason to be peculiar: the 9-th and 10-th landmarks are subject to a constraint. In the construction of the facial image, $y_9 - y_{10}$ is constant for all the vectors in the dataset (and equal to the value of this quantity in the reference portrait). For a similar reason, we have not included the segment 7–12 in figure 4 (main article), despite there being a strong oblique correlation involving both quantities, since this correlation reflects another *a priori* constraint in the dataset: the 12-th landmark height coincides with the mouth's height by con-

struction. $\mathsf{x}_{12} = \mathsf{x}_7 = \mathsf{x}_{18}$. Equivalently, in the case of inter-landmark distances (see table 3), the presence of the constraint $\mathsf{h} = 1$ induces a null eigenvalue in C and leads to negative correlations among some facial coordinates $d_{1,2,3,4}$, not directly interpretable, as with $\langle \mathsf{y}_9 \mathsf{x}_{10} \rangle$. The Maximum Entropy method can provide an interpretation of the results of the empirical matrix C, correcting the artifact induced by such constraints (or quasi-constraints) [1].

To summarise, the inter-landmark segment angles provide a clear interpretation of C, which, in its turn provide a cue of the most relevant angles that we evaluate when we form impressions about a face. To perform a rigorous assessment of the relative importance of various facial elements, however, one should use an inference technique going beyond the bare correlation.

14 Application of the Maximum Entropy method

The above arguments motivate a Maximum Entropy-based approach to the problem [6, 7]. The goal is to infer a probability distribution $\mathcal{L}(\mathbf{y})$ from the experimental dataset $\mathcal{S}$ (see 2). $\mathcal{L}$ reproduces by construction some data sufficient statistics (at least two-coordinate correlations). $\mathcal{L}(\mathbf{y})$ is a probabilistic generative model of the dataset $\mathcal{S}$, and can be interpreted as the probability of the facial image with facial coordinates $\mathbf{y}$ (and fixed reference portrait) of being sculpted by any subject (or by a given subject having selected or sculpted the dataset $\mathcal{S}$). Inferring $\mathcal{L}$, one also infers a matrix (or a tensor) of *effective interactions* between couples (or $p > 2$-plets) of facial coordinates, that reflect the relative influence of the facial feature ins each other. This approach provides a theoretical framework allowing to rigorously account for *a priori* correlations and constraints, and to address on information-theoretical grounds the relative relevance of various variables [1].

15 Higher order and spurious correlations

A natural and relevant question is to what extent higher-order correlations of the data are statistically significant. In other words, whether three-coordinate empirical correlations $\langle y_i y_j y_k \rangle$ in our dataset exhibit a large t-value. The answer is that, although we do observe non-negligible three-coordinate correlations, we cannot attribute a cognitive origin to them–they are rather generated by an artifact of the genetic algorithm. In experiments E1-3, the vectors of the initial population, $\mathbf{f}^{(s,n)}(0)$ (see sec. 3) exhibit small 2- and 3-distance correlations that self-propagate and grow through the generations. Indeed, different populations sculpted (after $T = 10$ generations) by the genetic null model (with random left-right choices) exhibit significant 2- and 3-distance correlations, $C^{(2,3)}_{\text{null}}$. The null correlations are to be "subtracted" from those of the experiments with human subjects, $C^{(2,3)}_{\text{obs}}$, in order to isolate relevant correlations of cognitive origin only ($C^{(2,3)}_{\text{h}}$). This is an interesting inference problem *per se*, of wide generality. It would arise also in experiments with natural facial images that are selected by subjects. The C_{null} correlations in this case would correspond to the background correlations corresponding to the database of natural images, prior to the selection by the subjects.

Subtraction of 3-order null correlations. On the one hand, 3-order correlation tensors $C^{(3)}_{\rm obs}$ and $C^{(3)}_{\rm null}$ coincide within their statistical errors. We interpret this fact concluding that our experimental scheme does not allow to elicit relevant 3-coordinate correlations of cognitive origin. Nevertheless, we believe that such high-order correlations may exist and play a role in the cognitive process of facial discrimination, and would probably emerge in larger experimental datasets, with higher values of S. **Subtraction of 2-order null correlations.** On the other hand, the 2-coordinate correlation matrix $C^{(2)}_{\rm obs}$ measured from the experimental data of E1,3 is clearly distinguishable from $C^{(2)}_{\rm null}$, and exhibits larger matrix elements in absolute value. The "subtraction" of null from observed correlations cannot be performed simply as $C^{(2)}_{\rm h} = C^{(2)}_{\rm obs} - C^{(2)}_{\rm null}$, since this leads to a non-positive definite matrix in general. An alternative method, that we will motivate, analyse and describe in detail in a forthcoming communication, is given by the following procedure: (i) the non-standarised connected correlation matrices corresponding to the null experiment and to the experiments with humans are first defined: $\tilde{C}_{{\rm null}ij} = \langle f_i f_j\rangle - \langle f_i\rangle\langle f_j\rangle$ and so with $\tilde{C}_{\rm obs}$; (ii) one then defines the *interaction matrices*: $J_{\rm obs} = \tilde{C}_{\rm obs}^{-1}$, $J_{\rm null} = \tilde{C}_{\rm null}^{-1}$; (iii) the interaction matrix $J_{\rm h}$ is defined in the following way:

$$J_{\rm h} = E_{\rm obs}{}^{\dagger}\,{\rm diag}(\boldsymbol{\epsilon}_{\rm h})\,E_{\rm obs} \tag{21}$$

$$\epsilon_{{\rm h}i} = \epsilon_{{\rm obs}i} - \left[E_{\rm obs} J_{\rm null} E_{\rm obs}{}^{\dagger}\right]_{ii} \tag{22}$$

where $E_{\rm obs}$ is the matrix diagonalising $J_{\rm obs}$ (or $\tilde{C}_{\rm obs}$) and $\boldsymbol{\epsilon}_{\rm h}$ are its eigenvalues; (iv) one defines $C_{\rm h} = J_{\rm h}{}^{-1}$; (v) finally, one standarises the matrix $C_{\rm h}$: $C_{ij} = C_{{\rm h}ij}\,(\epsilon_{{\rm h}i}\epsilon_{{\rm h}j})^{1/2}$. In steps (i-v) we have just lowered each eigenvalue of matrix $J_{\rm obs}$, $\epsilon_{{\rm obs}j}$, by a quantity which is the expected value of $J_{\rm null}$ according to the corresponding eigenvector of $J_{\rm obs}$. Since the effective null *interaction matrix* (in the language of Maximum Entropy inference) is much lower than the interactions of cognitive order, the D quantities $\epsilon_{{\rm h}i}$ are all positive. We have used this method to isolate the spurious and artifact correlations $C^{(2)}_{\rm null}$ from the observed experimental correlations $C_{\rm obs}$ in E1,E3, leading to the matrix called C throughout the article. The C matrix elements so obtained are very close to that of the matrix $C_{\rm obs} - C_{\rm null}$ (but the matrix C is positive definite). In Fig. 13 we show a comparison of $J_{\rm obs} - J_{\rm null}$ vs. $J_{\rm h}$, for which this comparison is more evident since these matrices are not subject to the standarisation constraint. This fact suggests that the method efficiently "removes" the spurious, artifact correlations induced by the genetic algorithm from the data. A definite confirmation will be provided in future experiments, in which the correlations present in the initial condition of the genetic algorithm will be removed. In any case, *all the results* presented in this article are qualitatively *identical* using simply $C = C_{\rm obs}$. In the future publication [1] we will present a further rigorous method to "subtract" $C_{\rm null}$ from $C_{\rm obs}$, motivated in the context of the Maximum Entropy method.

16 Bibliography

[1] M. Ibáñez-Berganza, M. Amico, and V. Loreto. In: *in preparation* (2019).

[2] Rainer Storn and Kenneth Price. "Differential Evolution – A Simple and Efficient Heuristic for global Optimization over Continuous Spaces". In: *Journal of Global Optimization* 11.4 (Dec. 1997), pp. 341–359. ISSN: 1573-2916. DOI: 10.1023/A:1008202821328. URL: https://doi.org/10.1023/A:1008202821328.

[3] Dimitris K. Tasoulis, Vassilis P. Plagianakos, and Michael N. Vrahatis. "Clustering in evolutionary algorithms to efficiently compute simultaneously local and global minima". In: *Evolutionary Computation, 2005. The 2005 IEEE Congress on.* Vol. 2. IEEE, 2005, pp. 1847–1854. URL: http://ieeexplore.ieee.org/abstract/document/1554912/ (visited on 09/29/2017).

[4] Harold Hill et al. "How Different is Different? Criterion and Sensitivity in Face-Space". In: *Frontiers in Psychology* 2 (2011), p. 41. ISSN: 1664-1078. DOI: 10.3389/fpsyg.2011.00041. URL: http://journal.frontiersin.org/article/10.3389/fpsyg.2011.00041.

[5] Tim Valentine, Michael B. Lewis, and Peter J. Hills. "Face-space: A unifying concept in face recognition research". In: *The Quarterly Journal of Experimental Psychology* 69.10 (2016), pp. 1996–2019. DOI: 10.1080/17470218.2014.990392. eprint: http://dx.doi.org/10.1080/17470218.2014.990392. URL: http://dx.doi.org/10.1080/17470218.2014.990392.

[6] E. T. Jaynes. "Information Theory and Statistical Mechanics". In: *Phys. Rev.* 106 (4 May 1957), pp. 620–630. DOI: 10.1103/PhysRev.106.620. URL: https://link.aps.org/doi/10.1103/PhysRev.106.620.

[7] H. Chau Nguyen, Riccardo Zecchina, and Johannes Berg. "Inverse statistical problems: from the inverse Ising problem to data science". In: *Advances in Physics* 66.3 (2017), pp. 197–261. DOI: 10.1080/00018732.2017.1341604. URL: https://doi.org/10.1080/00018732.2017.1341604.

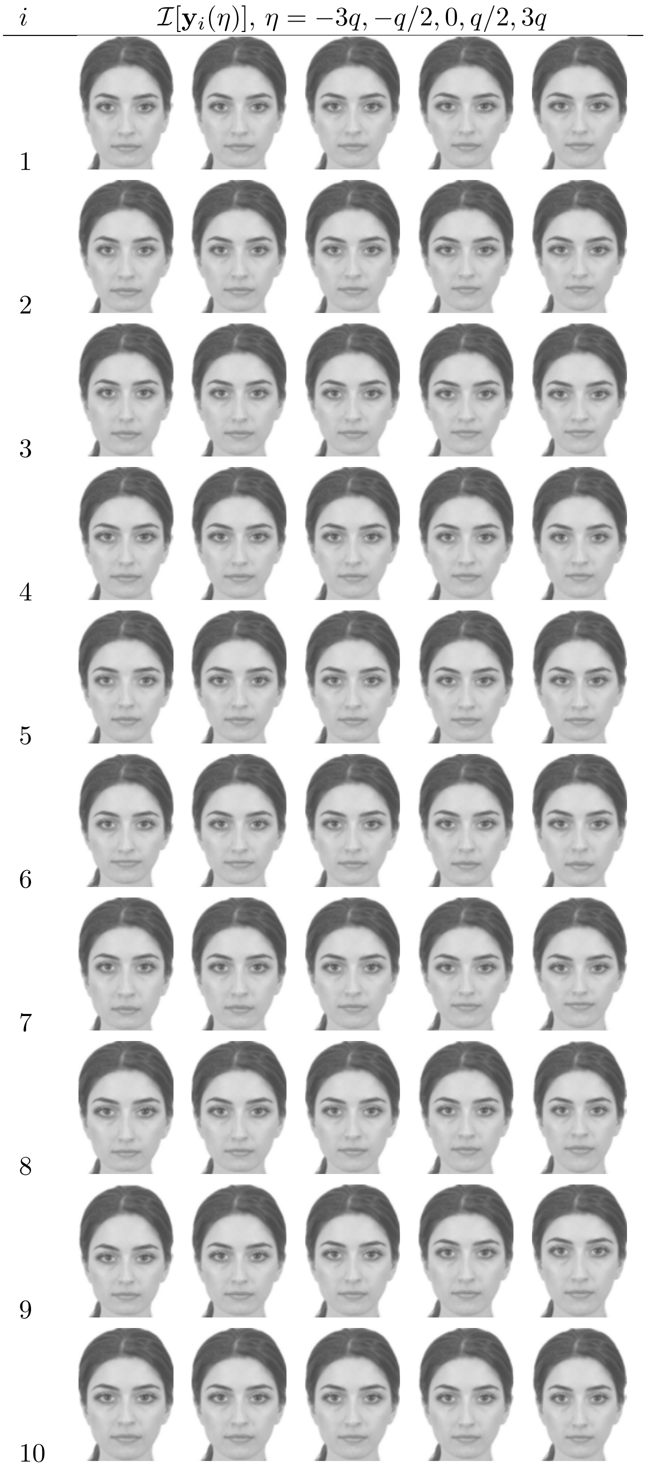

Figure 10: Facial images $\mathcal{I}[\eta\,\mathbf{e}^{(i)}]$ corresponding to various eigenvectors i (in different rows). Different columns correspond to various values of η, the central column ($\eta = 0$) is the average sculpted facial image in all rows. While high rows represent uncommon deformations (at a given η) with low C-eigenvalue λ_i, lower columns represent common deformations expanding most of the dataset variability.

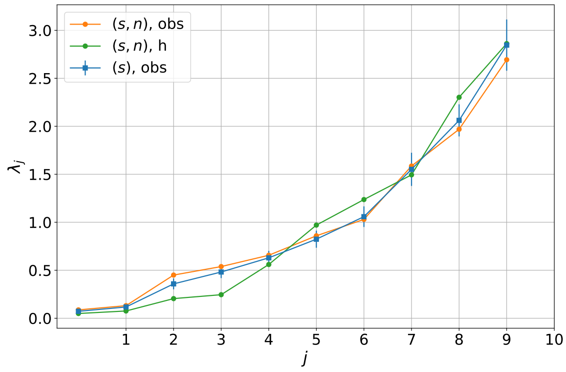

Figure 11: Spectrum of matrix C (blue curve). Larger than one eigenvalues correspond to eigenvectors that vary more than the physical (standarised) components, and vice-versa. The orange curve is the spectrum of the correlation matrix obtained as the average of $y_i^{(s,n)} y_j^{(s,n)}$ over both subject and population indices, (s, n). The green line is the spectrum of matrix C_{h} (see 15).

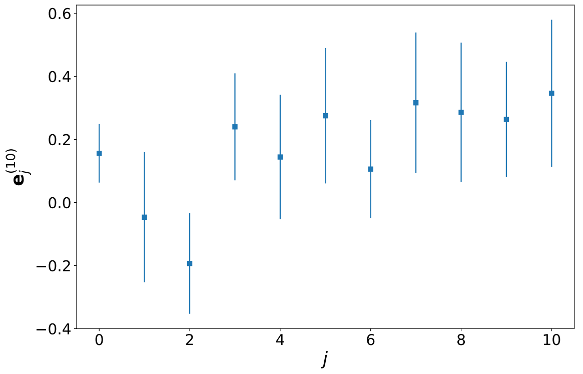

Figure 12: The vector components of the eigenvector $\mathbf{e}^{(10)}$ of matrix C (for inter-landmark distances), $e_j^{(10)}$, vs. j. The error-bars have been calculated by bootstrapping. The 10-th eigenvector (see figure 10) is essentially a scale transformation of the horizontal quantities (barely by the same factor, except for the inter-eye distance), and a linear combination of vertical quantities with smaller factors.

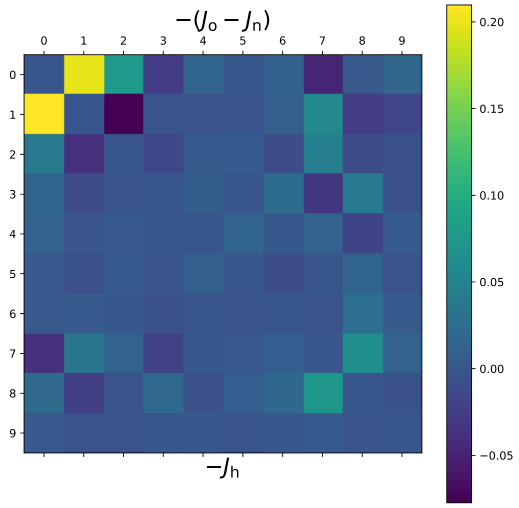

Figure 13: Comparison among the matrices $J_{\rm obs} - J_{\rm null}$ and $J_{\rm h}$ for Cartesian landmark coordinates. The upper triangle corresponds to the $-(J_{\rm obs} - J_{\rm null})$ matrix elements (the minus sign allows for a direct comparison with the C matrix). The lower triangle, to $-J_{\rm h}$ matrix elements. The diagonal has been set to zero for a clearer comparison.
